# Response of spatially defined microglia states with distinct chromatin accessibility in a mouse model of Alzheimer’s disease

**DOI:** 10.1038/s41593-025-02006-0

**Published:** 2025-07-14

**Authors:** Alberto Ardura-Fabregat, Lance Fredrick Pahutan Bosch, Emile Wogram, Omar Mossad, Roman Sankowski, Philipp Aktories, Lina Kieger, James Cook, Dilara Hasavci, Hatice Ulupinar, Daniel Brock, Fang Wang, Nicola Iovino, Samuel Wald, Sebastian Preissl, Bahtiyar Yilmaz, Daniel Schnepf, Andrew J. Macpherson, Thomas Blank, Katrin Kierdorf, Marco Prinz

**Affiliations:** 1 Institute of Neuropathology, Faculty of Medicine, University of Freiburg, Freiburg, Germany; 2 Faculty of Biology, University of Freiburg, Freiburg, Germany; 3 Berta-Ottenstein-Programme for Clinician Scientists, Faculty of Medicine, University of Freiburg, Freiburg, Germany; 4 Max Planck Institute of Immunobiology and Epigenetics (MPI-IE), Freiburg, Germany; 5 Centre for Integrative Biological Signalling Studies (CIBSS), University of Freiburg, Freiburg, Germany; 6 Institute of Experimental and Clinical Pharmacology and Toxicology, Faculty of Medicine, University of Freiburg, Freiburg, Germany; 7 Institute of Pharmaceutical Sciences, Pharmacology & Toxicology, University of Graz, Graz, Austria; 8 Field of Excellence BioHealth, University of Graz, Graz, Austria; 9 Department of Visceral Surgery and Medicine, Bern University Hospital, University of Bern, Bern, Switzerland; 10 Maurice Müller Laboratories, Department for Biomedical Research, University of Bern, Bern, Switzerland; 11 Institute of Virology, Faculty of Medicine, University of Freiburg, Freiburg, Germany; 12 Spemann Graduate School of Biology and Medicine, Albert Ludwigs University Freiburg, Freiburg, Germany; 13 Signalling Research Centres BIOSS and CIBSS, University of Freiburg, Freiburg, Germany

**Keywords:** Neuroimmunology, Innate immune cells

## Abstract

Microglial spatial heterogeneity remains a crucial yet not fully answered question in the context of potential cell-directed therapies for Alzheimer’s disease (AD). There is an unclear understanding of the dynamics of distinct microglia states adjacent to or far from amyloid-beta (Aβ) plaques and their contributions to neurodegenerative diseases. Here we combine multicolor fluorescence cell fate mapping, single-cell transcriptional analysis, epigenetic profiling, immunohistochemistry and computational modeling to comprehensively characterize the relation of plaque-associated microglia (PAM) and non-plaque-associated microglia (non-PAM) in a mouse model of AD. We show that non-PAM are a distinct and highly dynamic microglial state, transitioning to PAM after Aβ plaque deposition in female mice. Non-PAM modulate the cell population expansion in response to amyloid deposition and rapidly respond to environmental cues. Indeed, Csf1 signaling modulates non-PAM-to-PAM transition during disease progression. Our data suggest that microglia states and their dynamics between each other can have distinct contributions to disease, and they may be targeted for the treatment of AD.

## Abstract (web summary)

The dynamics of microglia states adjacent to or far from amyloid-beta plaques are unclear. Here the authors show that non-plaque-associated microglia modulate the cell population expansion in response to amyloid deposition, and Csf1 signaling regulates their transition to the amyloid-associated state.

## Main

As tissue-resident macrophages of the central nervous system parenchyma, microglia are not only involved in tissue development and homoeostasis but also in virtually all neuroinflammatory, neurodegenerative, neuropsychiatric and neuro-oncological disorders^1^. This functional engagement is accompanied by strong microglial reactivity and expansion of differentiated preexisting microglia, called microgliosis^2,3^.

Adult microglia are self-maintained^4^ and originate from prenatal macrophage progenitors from the embryonic yolk sac^5–7^. Recent transcriptomic studies revealed phenotypic and functional heterogeneity of microglia across development, steady state and disease^8–10^, giving insights into the heterogeneous and dynamic states of microglia. However, the relative functional contribution of these microglial states to neuronal damage and regeneration in neurodegenerative disorders such as AD remain to be fully explored.

Recent sophisticated single-cell analyses recognized distinct microglial states in AD-like mouse models^11–13^ or AD in humans^14–16^. Based on their transcriptomic identity, disease-associated microglia (DAM) were described among these cellular states and correlated with plaque-associated microglial states^11^. Similar to DAM, a microglial neurodegenerative state (MgnD) in the context of microglia activation during neurodegeneration was defined^17^. AD-associated DAM/MgnD exhibited a noteworthy gene signature with high expression levels, including *Itgax* (integrin subunit alpha x), *Clec7a* (C-type lectin domain containing 7A), *Trem2* (triggering receptor expressed on myeloid cells 2), *Apoe* (apolipoprotein E), *Lpl* (lipoprotein lipase) and *Cst7* (cystatin 7). Trem2-dependent signaling was highlighted as an essential component for neurodegeneration-associated microglial clustering and limitation of AD pathology^18–20^.

Although these important studies highlighted context-associated microglial states in AD, they addressed neither their cellular kinetics nor their functional and spatial relationship to non-PAM. Furthermore, to what extent distinct microglial states are differentially modulated during disease—for example, by environmental factors or age—and the effects on disease pathogenesis remained unclear.

In the present study, we comprehensively characterized the distribution, kinetics, gene expression profiles and differential fates of PAM and non-PAM subsets during AD-related neurodegeneration. By combining in vivo expansion mapping systems, massively parallel single-cell RNA sequencing (scRNA-seq) and epigenetic profiling, we revealed a dynamic and sensitive adaptation process by which non-PAM respond to amyloid pathology, producing new PAM. This gradual transition between microglia states offers a potential therapeutic intervention window to modulate the fate of microglia during neurodegeneration and ameliorate disease pathology.

## Results

### Non-PAM develop to clonally expanding PAM at amyloid plaques

**Fig. 1:**
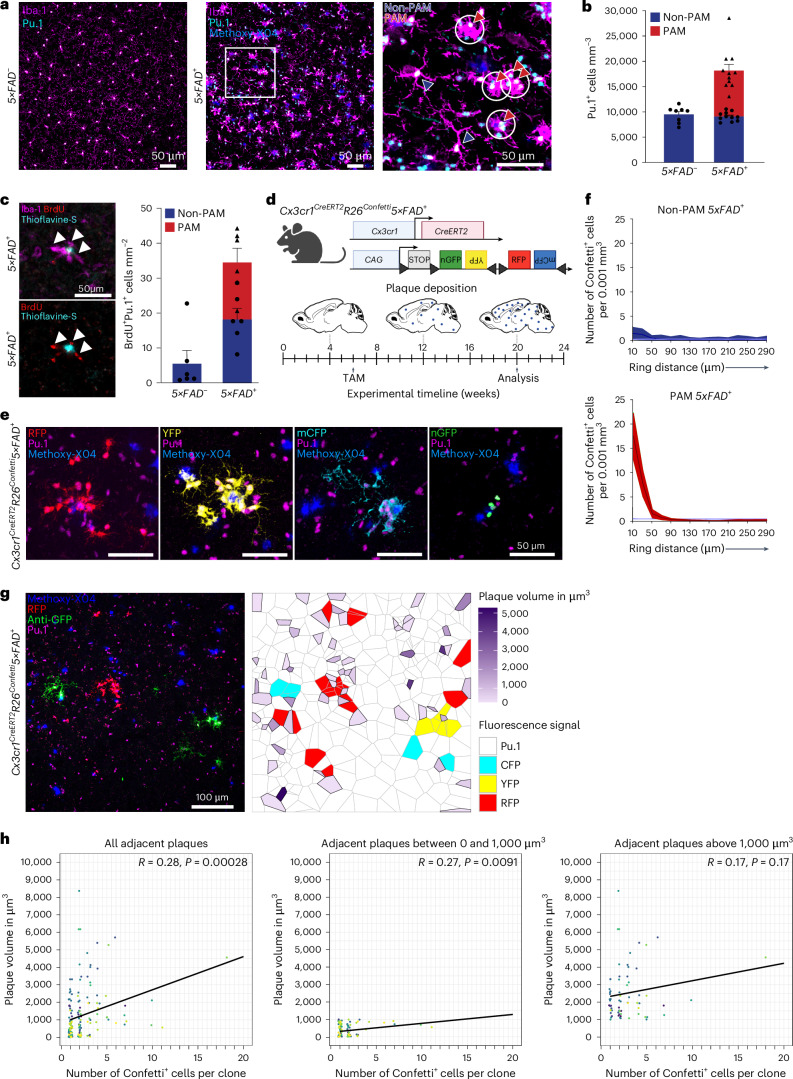
PAM clonally expand at amyloid plaques. **a**, Representative images of *5×FAD*^*−*^ (left) and *5×FAD*^*+*^ animals (center, right). Iba-1 (magenta), Methoxy-X04 (blue) and Pu.1 (cyan) are shown. Right: depiction of Pu.1^+^Iba-1^+^ PAM (red arrowheads) and Pu.1^+^Iba-1^+^ non-PAM (blue arrowheads). White circles indicate 30-µm ring around amyloid plaques. Cells with direct contact to plaques were defined as PAM. Scale bars, 50 µm. **b**, Quantification of Pu.1^+^ microglia per mm^3^ separated in PAM (red, individual mice: triangles) and non-PAM (blue, individual mice: circles) in *5×FAD*^*+*^ (*n* = 11) and *5×FAD*^*−*^ (*n* = 8) mice. Mean ± s.e.m. is shown. **c**, Left: representative images of *5×FAD*^*+*^ mice treated with BrdU. BrdU (red), Iba-1 (magenta) and thioflavine-S (cyan) are shown. Arrowheads indicate BrdU^+^Iba-1^+^ PAM. Scale bar, 50 µm. Right: quantification of BrdU^+^Pu.1^+^ PAM (red, individual mice: triangles) and non-PAM (blue, individual mice: circles) per mm^2^ in *5×FAD*^*+*^ (*n* = 6) and *5×FAD*^*−*^ (*n* = 6) mice. Mean ± s.e.m is shown. **d**, Experimental scheme of *Cx3cr1*^*CreERT2*^*R26*^*Confetti*^*5×FAD*^*+*^ animals. Created with BioRender.com. **e**, Representative images from *Cx3cr1*^*CreERT2*^*R26*^*Confetti*^*5×FAD*^*+*^ animals. Confetti^+^ microglia clones were found as nGFP (green), cytoplasmic YFP (yellow), RFP (red) or mCFP (cyan). Pu.1 (magenta) and Methoxy-X04 (blue) are shown. Scale bars, 50 µm. **f**, MC simulation: densities of Confetti^+^ non-PAM (blue, top) and PAM (red, bottom) of *Cx3cr1*^*CreERT2*^*R26*^*Confetti*^*5×FAD*^*+*^ animals (*n* = 11) shown relative to randomized datasets (purple). Densities are displayed relative to measured distances (that is, ring distance (µm)). Mean and 98% confidence intervals are shown. **g**, Left: representative image of Confetti^+^ PAM clones in *Cx3cr1*^*CreERT2*^*R26*^*Confetti*^*5×FAD*^*+*^ animals. anti-GFP (green), anti-RFP (red), Methoxy-X04 (blue) and Pu.1 (magenta) are shown. Scale bar, 100 µm. Right: representative Voronoi grid of plaque sizes and Confetti^−^Pu.1^+^ and Confetti^+^Pu.1^+^ microglia. Confetti^−^Pu.1^+^ (white), CFP^+^Pu.1^+^ (cyan), YFP^+^Pu.1^+^ (yellow) and RFP^+^Pu.1^+^ (red) microglia are shown. Volume of Methoxy-X04^+^ plaques is color coded as indicated in the legend. **h**, Correlations between Confetti^+^ microglia clone size and adjacent Methoxy-X04^+^ plaques. Colors of individual points represent separately analyzed images (*N* = 17) across individuals (*n* = 4). Black line indicates linear regression. Left: correlation for all Methoxy-X04^+^ plaque sizes (R = 0.28, ***P *=* 0.00028). Middle: correlation for <1,000-µm^3^-sized Methoxy-X04^+^ plaques (*R* = 0.27, **P *=* 0.0091). Right: correlation for Methoxy-X04^+^ plaques >1,000 µm^3^ (*R* = 0.17, NS *P* *=* 0.17). NS, not significant. Source data

To explore spatial heterogeneity of microglia during neurodegeneration, we analyzed the distribution of Pu.1^+^ microglia in the frontal cortices of 44-week-old female *5×FAD*^*+*^ animals (Fig. 1a). Compared to homogenously distributed microglia in the non-transgenic *5×FAD*^*−*^ mice, microglia in transgenic *5×FAD*^+^ animals showed an aberrant bimodal distribution. In fact, Pu.1^+^Iba-1^+^ microglia existed as either PAM, in direct physical contact to Methoxy-X04-labeled amyloid deposits and their cell bodies within a 30-µm radius of said deposits, or as non-PAM, ramified cells distributed at greater distance from—and with no contact to—deposited amyloid. Quantification of Pu.1^+^ PAM and non-PAM showed a clear PAM expansion in *5×FAD*^*+*^ animals but similar numbers of non-PAM compared to controls (Fig. 1b). Assessment of proliferation capacity by 5-bromo-2′-deoxyuridine (BrdU) incorporation revealed a high proliferative capacity of PAM and non-PAM in *5×FAD*^*+*^ mice, whereas microglia in *5×FAD*^*−*^ mice incorporated BrdU to a lesser extent (Fig. 1c).

To study cell dynamics in PAM and non-PAM over time and to distinguish spatially distributed microglia and their progeny, we generated *Cx3cr1*^*CreERT2*^*R26*^*Confetti*^*5×FAD*^*+*^ mice that randomly label individual microglia with nuclear green fluorescent protein (nGFP), cytoplasmic yellow fluorescent protein (YFP), cytoplasmatic red fluorescent protein (RFP) or membrane-tagged cyan fluorescent protein (mCFP) upon tamoxifen (TAM) application (Fig. 1d,e). Notably, in *5×FAD*^*+*^ mice, same-colored Confetti^+^Pu.1^+^ PAM clusters were found around Methoxy-X04^+^ amyloid deposits (Fig. 1e,f). To understand the expansion dynamics, such as clonal growth^21,22^, within the PAM and non-PAM population, the proximities of each Confetti^+^ microglia to same-colored cells in space were determined and compared to a randomly generated dataset based on a Monte Carlo (MC) simulation^22^. Whereas PAM showed non-clonal random expansion, PAM clonally expand at the plaque site (Fig. 1g). We next tested for potential correlation of clone sizes with amyloid plaque dimensions by generating Voronoi grids of amyloid plaques and adjacent Confetti^+^Pu.1^+^PAM clones to visualize this spatial relationship (Fig. 1g). Clone dimensions of PAM were overall highly correlative to amyloid plaque size (Fig. 1h). However, amyloid plaques of greater than 1,000 µm^3^ showed no further correlation to the Confetti^+^Pu.1^+^ PAM clone size, indicating that progressive amyloid deposition outcompetes clonal growth of PAM.

**Fig. 2:**
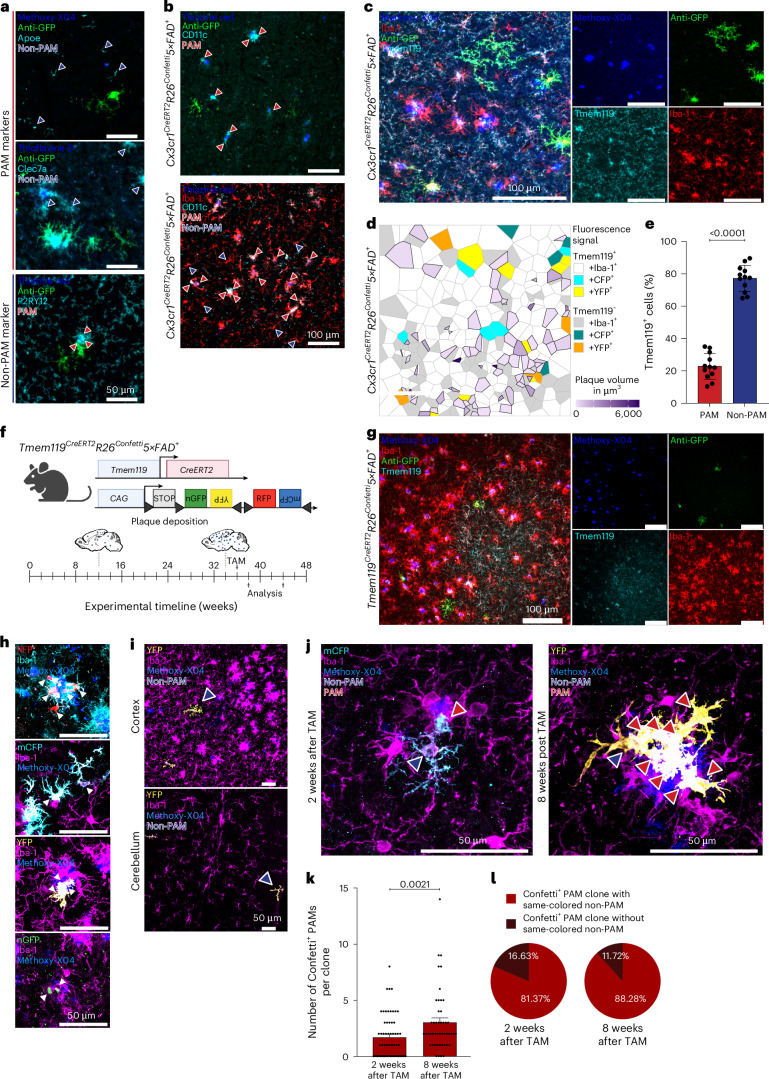
Individual non-PAM give rise to clones of expanding PAM at the plaque sites. **a**, Representative images of Confetti^+^ microglia in *Cx3cr1*^*CreERT2*^*R26*^*Confetti*^*5×FAD*^*+*^ mice. Immunofluorescence for anti-GFP (green) and thioflavine-S/thiazine red/Methoxy-X04 (blue) are shown with the following markers (cyan). Top: APOE and CLEC7A. Bottom: P2RY12. Blue arrowheads indicate non-PAM; red arrowheads highlight PAM. Scale bars, 50 µm. **b**, Top: representative images for anti-GFP (green) and thiazine red (blue) together with CD11c (cyan). Bottom: images of Iba-1^+^CD11c^+^ PAM restricted to amyloid plaques in *Cx3cr1*^*CreERT2*^*R26*^*Confetti*^*5×FAD*^*+*^ animals. Thiazine red (blue), Iba-1 (red) and CD11c (cyan) are shown. Blue arrowheads indicate non-PAM and red arrowheads indicate CD11c^+^ PAM. Scale bars, 100 µm. **c**, Left: illustrative image of Confetti^+^ microglia in *Cx3cr1*^*CreERT2*^*R26*^*Confetti*^*5×FAD*^*+*^ animals. Iba-1 (red), anti-GFP (green), Methoxy-X04 (blue) and Tmem119 (cyan) are shown. Scale bars, 100 µm. Right: individual channels are shown. **d**, Voronoi grid visualizing plaque sizes and the positioning of Confetti^−^ and Confetti^+^ Tmem119^+^Iba-1^+^ microglia (non-PAM) and Confetti^−^ and Confetti^+^ Tmem119^−^Iba-1^+^ microglia (PAM) in 20-week-old *Cx3cr1*^*CreERT2*^*R26*^*Confetti*^*5×FAD*^*+*^ mice. Tmem119^+^Iba-1^+^ (white), CFP^+^Tmem119^+^Iba-1^+^ (cyan), YFP^+^Tmem119^+^Iba-1^+^ (yellow), Tmem119^−^Iba-1^+^ (gray), CFP^+^Tmem119^−^Iba-1^+^ (green) and YFP^+^Tmem119^−^Iba-1^+^ (orange) microglia are shown. Volume of Methoxy-X04^+^ plaques is color coded as indicated in the legend. **e**, Quantification of Tmem119^+^ PAM (red) and non-PAM (blue) in *Cx3cr1*^*CreERT2*^*R26*^*Confetti*^*5×FAD*^*+*^ mice (*n* = 12). Dots represent individual animals. Mean ± s.e.m. is shown. ***P *<* 0.0001. **f**, Experimental scheme of *Tmem119*^*CreERT2*^*R26*^*Confetti*^*5×FAD*^*+*^ mice. Scheme was created with BioRender.com. **g**, Characteristic images of *Tmem119*^*CreERT2*^*R26*^*Confetti*^*5×FAD*^*+*^ animals 8 weeks after TAM application. Images are shown for Methoxy-X04 (blue), anti-GFP (green), Tmem119 (cyan) and Iba-1 (red). Scale bars, 100 µm. Data are shown from one independent experiment. **h**, Characteristic images of Confetti^+^Iba-1^+^ microglia (arrowheads) clones at amyloid plaques in *Tmem119*^*CreERT2*^*R26*^*Confetti*^*5×FAD*^*+*^ animals 8 weeks after TAM application. Clones are found in all Confetti colors: RFP (red), mCFP (cyan), YFP (yellow) and nGFP (green). Immunofluorescence for Iba-1 (cyan, top) and magenta (middle and bottom) and Methoxy-X04 (blue) are shown. Scale bars, 50 µm. Data are shown from one independent experiment. **i**, Typical confocal pictures of single labeled Confetti^+^Iba-1^+^ non-PAM (blue arrowheads) in the cortex (top) and cerebellum (bottom) of 44-week-old *Tmem119*^*CreET2R*^*R26*^*Confetti*^*5×FAD*^*+*^ animals 8 weeks after TAM application. Immunofluorescence for Iba-1 (magenta), Methoxy-X04 (blue) and YFP (yellow). Scale bars, 50 µm. Data are shown from one independent experiment. **j**, Representative images from *Tmem119*^*CreERT2*^*R26*^*Confetti*^*5×FAD*^*+*^ animals 2 weeks (left) or 8 weeks (right) after TAM application. Confetti labeling was observed in both Iba-1^+^ (magenta) non-PAM (blue arrowheads) and Iba-1^+^ PAM (red arrowheads). Immunofluorescence for Methoxy-X04 (blue), YFP (yellow) and mCFP (cyan) is shown. Scale bars, 50 µm. **k**, Quantification of same-colored Confetti^+^ PAM per clone in *Tmem119*^*CreER*^*R26R*^*Confetti*^*5×FAD*^*+*^ animals 2 weeks (*N* = 62) and 8 weeks (*N* = 48) after TAM application. Mean ± s.e.m. is shown. Symbols represent individual clones from biological replicates (*n* = 2). *P* *=* 0.0021. **l**, Quantification of the percentage of same-colored Confetti^+^ PAM clones that were either associated (red) or not associated (dark red) with an individual same-colored Confetti^+^ non-PAM. Pie charts represent distribution at 2 weeks (left) and 8 weeks (right) after TAM injection. Source data

**Extended Data Fig. 1:**
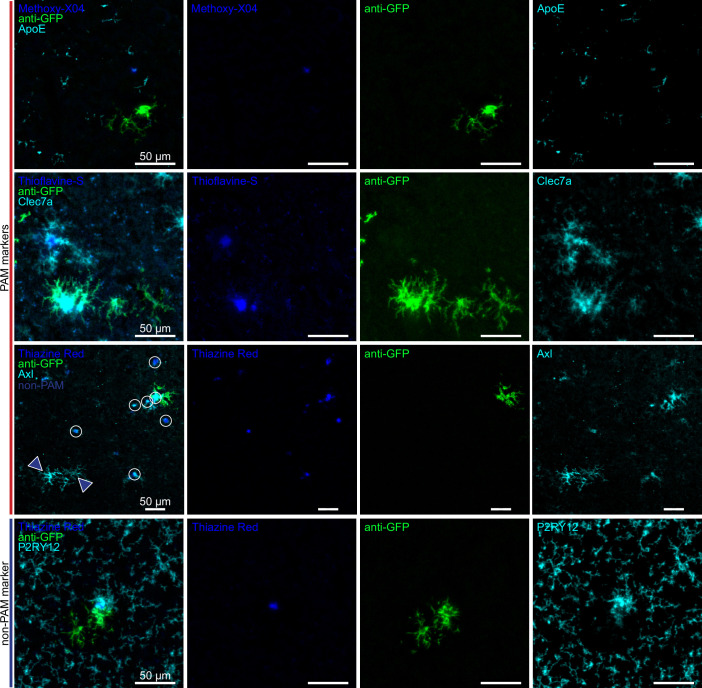
Tracing of PAM and non-PAM expansion dynamics during amyloid pathology in *Cx3cr1*^*CreERT2*^*R26*^*Confetti*^*5xFAD*^*+*^ animals. Illustrative confocal images of Confetti^+^ microglia in 20-week-old *Cx3cr1*^*CreERT2*^*R26*^*Confetti*^*5xFAD*^*+*^ animals. Immunofluorescence for anti-GFP (Confetti^+^, green), and thioflavine-S/Thiazine Red/Methoxy-X04 (blue) are depicted together with the following markers (cyan): upper three rows: ApoE, Clec7a (higher magnification and individual channels from Fig. 2) and Axl; lower row: P2RY12 (higher magnification and individual channels from Fig. 1). Arrowheads indicate non-PAM labelled by Axl. Scale bars = 50 µm.

To determine reliable markers to distinguish non-PAM and PAM beyond their distinct location, we tested previously reported DAM^11^ and MgnD^17^ markers such as *CD11c*, *Apoe*, receptor tyrosine kinase *Axl*, *Clec7a* and homeostatic microglia markers such as transmembrane protein 119 (*Tmem119*) and purinergic receptor *P2ry12*. For CLEC7A, APOE, AXL AND P2RY12, we found no clear separation between PAM and non-PAM (Fig. 2a and Extended Data Fig. 1a). In contrast, CD11c expression was highly restricted to PAM and virtually absent in non-PAM (Fig. 2b), representing a reliable marker for PAM. Similarly, Tmem119 revealed a strong labeling of non-PAM, whereas PAM showed only a minor expression of Tmem119 (Fig. 2c–e).

To interrogate the dynamic relationship between non-PAM and PAM, we took advantage of the selectively higher Tmem119 expression on non-PAM and generated *Tmem119*^*CreERT2*^*R26*^*Confetti*^*5×FAD*^*+*^ animals (Fig. 2f). We induced recombination in 36-week-old *Tmem119*^*CreERT2*^*R26*^*Confetti*^*5×FAD*^*+*^ animals. After 8 weeks, we detected Confetti^+^ microglia in both Tmem119^+^Iba-1^+^ non-PAM and Tmem119^−^Iba-1^+^ PAM, suggesting that initially labeled non-PAM gave rise to PAM at adjacent amyloid plaques (Fig. 2g,h). As expected, single labeled ramified Confetti^+^Iba-1^+^ non-PAM were found distant to amyloid plaques and in unaffected brain regions (Fig. 2i). Clusters of Confetti^+^Iba-1^+^ PAM were observed around cortical amyloid deposits in *Tmem119*^*CreERT2*^*R26*^*Confetti*^*5×FAD*^*+*^ mice, and the cell number per clone increased from 2 weeks to 8 weeks after TAM injection (Fig. 2j and Supplementary Videos 1 and 2). Notably, single Confetti^+^ non-PAM were always located adjacent to same-colored Confetti^+^ PAM clones (Fig. 2j and Supplementary Videos 1 and 2), and the percentage of PAM clones associated with an adjacent same-colored non-PAM remained stable over time (Fig. 2k,l). These data point to non-PAM as a highly versatile microglial population during neurodegeneration that dynamically respond to progressing amyloid pathology by giving rise to clonally expanding PAM.

### Peripheral stimuli shape PAM clonality in early disease

After establishing the dynamic transition of non-PAM to PAM during neurodegeneration, we examined whether clonal expansion of PAM can be modulated by environmental factors. Environmental factors such as gut microbiota and infections, as well as age, have been described to modify microglial behavior during amyloid pathology in mice^23,24^.

**Fig. 3:**
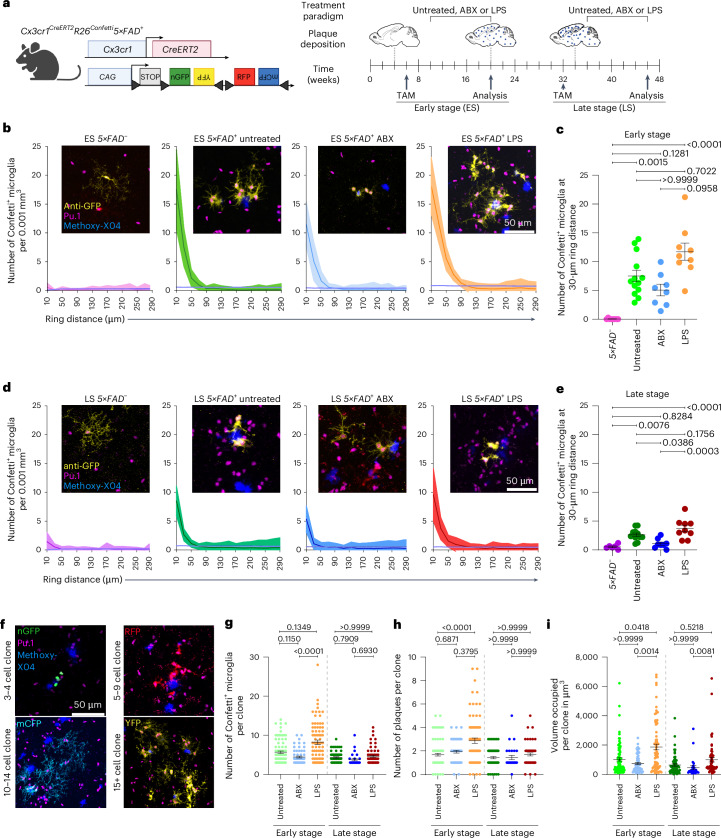
Microglial clonality around amyloid plaques is modulated by peripheral stimuli, gut microbiota and disease stage. **a**, Experimental scheme for treatment paradigms in *Cx3cr1*^*CreERT2*^*R26*^*Confetti*^*5×FAD*^*+*^ animals and controls. Created with BioRender.com. **b**, MC simulation: densities of Confetti^+^ microglia in untreated *Cx3cr1*^*CreERT2*^*R26*^*Confetti*^*5×FAD*^*−*^ control littermates (pink, *n* = 8) and *Cx3cr1*^*CreERT2*^*R26*^*Confetti*^*5×FAD*^+^ animals that were either untreated (green, *n* = 13) or systemically treated with ABX (blue, *n* = 8) or LPS (orange, *n* = 9) at early stage, relative to randomized datasets (purple). Densities are displayed relative to measured distances (that is, ring distance (µm)). Mean and 98% confidence intervals are shown. Insets: representative images of anti-GFP-labeled Confetti^+^ (yellow) Pu.1^+^ (magenta) microglia in relation to Methoxy-X04 (blue). Scale bar, 50 µm. **c**, Quantification of Confetti^+^ cells located in a 30-µm ring distance. Each symbol represents one animal. Mean ± s.e.m. is shown. Specific *P* values of statistical tests are indicated. **d**, MC simulation: densities of Confetti^+^ microglia in untreated *Cx3cr1*^*CreERT2*^*R26*^*Confetti*^*5×FAD*^−^ control littermates (dark pink, *n* = 6) and *Cx3cr1*^*CreERT2*^*R26*^*Confetti*^*5×FAD*^+^ animals that were either untreated (dark green, *n* = 11) or systemically treated with ABX (dark blue, *n* = 8) or LPS (red, *n* = 9) at late stage, relative to randomized datasets (purple). Densities are displayed relative to measured distances (that is, ring distance (µm)). Means and 98% confidence intervals are shown. Representative images of anti-GFP-labeled Confetti^+^ (yellow) Pu.1^+^ (magenta) microglia in relation to Methoxy-X04 (Aβ, blue) are shown for each group. Scale bar, 50 µm. **e**, Quantification of Confetti^+^ cells located in a 30-µm ring distance. Each symbol represents one animal. Mean ± s.e.m. is shown. Specific *P* values of statistical tests are indicated. **f**, Representative images of same-colored Confetti^+^ clone sizes of Pu.1^+^ (magenta) microglia in *Cx3cr1*^*CreERT2*^*R26*^*Confetti*^*5×FAD*^+^ animals: 3–4 nGFP^+^ (green) cells; 5–9 RFP^+^ (red) cells; 10–14 mCFP^+^ (cyan) cells; and 15 or more YFP^+^ (yellow) cells. Methoxy-X04 (blue) is shown for amyloid plaques. Scale bar, 50 µm. **g**, Quantification of Confetti^+^ microglial cells per clone. Each dot represents one clone. Clones were analyzed for each group across all animals (*Cx3cr1*^*CreERT2*^*R26*^*Confetti*^*5×FAD*^+^ animals that were either untreated (green, *n* = 13, *N* = 87) or systemically treated with ABX (blue, *n* = 8, *N* = 62) or LPS (orange, *n* = 9, *N* = 79) at early stage and untreated *Cx3cr1*^*CreERT2*^*R26*^*Confetti*^*5×FAD*^−^ animals (dark pink, *n* = 6, *N* = 79) and *Cx3cr1*^*CreERT2*^*R26*^*Confetti*^*5×FAD*^+^ animals that were either untreated (dark green, *n* = 11, *N* = 61) or systemically treated with ABX (dark blue, *n* = 8, *N* = 27) or LPS (red, *n* = 9, *N* = 57) at late stage). Mean ± s.e.m. is shown. Specific *P* values of statistical tests are indicated. **h**, Quantification of the number of associated plaques per Confetti^+^ clone. Each dot represents one clone. Clones were analyzed for each group across all animals as described in **g**. Mean ± s.e.m. is shown. Specific *P* values of statistical tests are indicated. **i**, Quantification of volume occupied per clone in all groups analyzed. Each dot represents one clone. Clones were analyzed for each group across all animals as described in **g**. Mean ± s.e.m. is shown. Specific *P* values of statistical tests are indicated. ES, early stage; LS, late stage. Source data

Therefore, we treated *Cx3cr1*^*CreERT2*^*R26*^*Confetti*^*5×FAD*^*+*^ mice peripherally with low-dose lipopolysaccharide (LPS) to model low-grade peripheral inflammation or, alternatively, with antibiotics (ABX) to mimic loss of host microbiota at early stage or late stage of disease (Fig. 3a).

Clonal expansion of PAM at the plaque site occurred with different dynamics in young (Fig. 3b,c) and aged (Fig. 3d,e) *Cx3cr1*^*CreERT2*^*R26*^*Confetti*^*5×FAD*^*+*^ animals. PAM clone expansion was more prominent in young than aged *5×FAD*^*+*^ mice, pointing to different expansion dynamics at different stages of disease. Notably, clonal accumulation of Confetti^+^ PAM in transgenic mice at early stage was slightly reduced upon ABX treatment, whereas LPS application increased the expansion (Fig. 3b,c). In contrast, the clonal accumulation of Confetti^+^ PAM at the plaque site was minimally affected by either treatment paradigm in the late stage (Fig. 3d,e). Accordingly, we detected that PAM clone sizes, number of clone-associated plaques as well as clone territory were enhanced upon LPS challenge, but not in the absence of microbiota, at the early stage of disease (Fig. 3f–i). During the late stage of disease, no alteration of clone size was observed across the different treatment groups (Fig. 3f–i).

**Extended Data Fig. 2:**
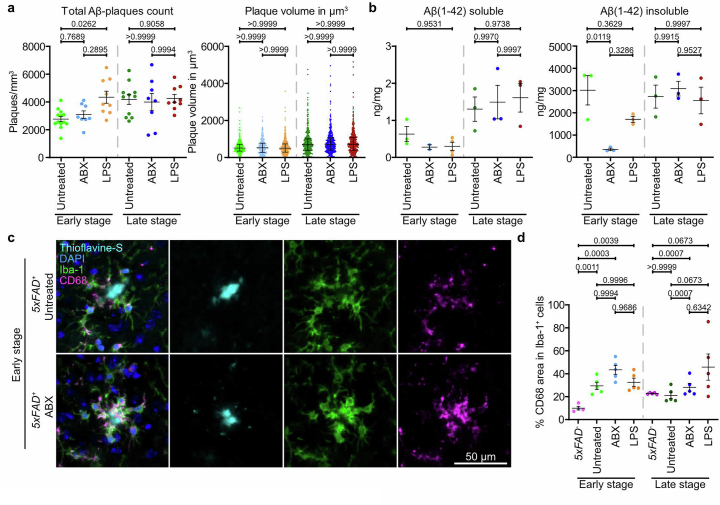
Minor modulation of plaque load by peripheral stimuli and gut microbiota. **(a)** Quantification of plaque number (left) and individual plaque sizes (right) of groups as shown in Fig. 3. Left: Each symbol represents one animal. Mean ± s.e.m. is shown. Right: Each symbol represents one analyzed plaque; N = 1,838 – 2,972 plaques per group. Median ± interquartile range is shown. Specific *P* values of statistical tests are indicated. **(b)** ELISA of human Aβ(1-42) peptides in soluble (left panel) and insoluble (right panel) fractions of cortices from untreated, ABX- and LPS-treated *Cx3cr1*^*CreERT2*^*R26*^*Confetti*^*5xFAD*^*+*^ animals at an early (untreated: n= 3, ABX: n=2, LPS: n = 4) and late stage (untreated: n= 3, ABX: n=3, LPS: n = 3) of disease. Each symbol represents one animal. Mean ± s.e.m. is shown. Specific *P* values of statistical tests are indicated. **(c)** Representative images of CD68^+^ PAM in untreated (upper panel) or ABX-treated (lower panel) *Cx3cr1*^*CreERT2*^*R26*^*Confetti*^*5xFAD*^*+*^ animals at an early stage of disease. Immunofluorescence for CD68 (magenta), Thioflavine-S (amyloid beta, cyan), Iba-1 (microglia, green) and DAPI (blue) is shown. Scale bar = 50 µm. **(d)** Quantification of the percentage of CD68^+^ area in Iba-1^+^ microglial cell bodies of untreated, ABX- and LPS-treated *Cx3cr1*^*CreERT2*^*R26*^*Confetti*^*5xFAD*^*+*^ animals at an early and late stage of pathology (all groups: n= 5) compared to untreated control littermates (n =4). Each symbol represents one animal. Mean ± s.e.m. is shown.Specific *P* values of statistical tests are indicated. Source data

We next examined whether modulation of PAM clonality was accompanied by altered amyloid pathology. Only subtle changes in the median plaque size were detectable upon any treatment in the early-stage and late-stage groups, despite a significantly increased number of plaques after LPS treatment in the early stage (Extended Data Fig. 2a). However, ABX-treated and LPS-treated groups at the early stage showed a slight decrease in levels of soluble and insoluble Aβ_1–42_ (Extended Data Fig. 2b). Intriguingly, ABX treatment enhanced phagocytosis by PAM during the early stage, yet no changes were detectable in the late stage (Extended Data Fig. 2c,d). Collectively, these data suggest that the kinetics of PAM clonality is dependent on the duration of the disease course.

### Non-PAM remain responsive and plastic during amyloid pathology

**Fig. 4:**
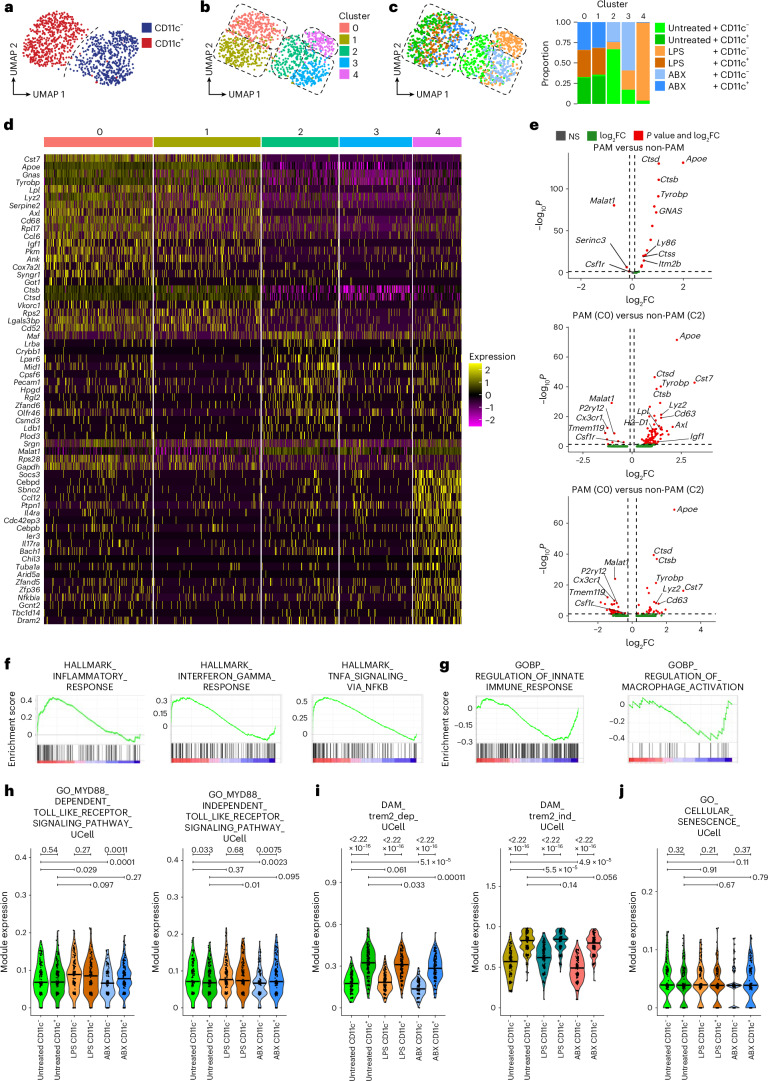
Non-PAM show transcriptional plasticity toward peripheral stimuli and gut dysbiosis at early stages of amyloid pathology. **a**, UMAP plot visualizing CD11c^−^ non-PAM (blue) and CD11c^+^ PAM (red) from untreated, LPS-treated and ABX-treated *Cx3cr1*^*CreERT2*^*R26*^*Confetti*^*5×FAD*^*+*^ animals at an early stage of disease. Each dot represents one cell (*N* = 1,095). **b**, UMAP plot of cell clusters (C0–C4) identified by unsupervised clustering of all analyzed cells from untreated, LPS-treated and ABX-treated *Cx3cr1*^*CreERT2*^*R26*^*Confetti*^*5×FAD*^*+*^ animals at an early stage of disease. Each color represents one cell cluster; each cluster is outlined by dotted lines. Each dot represents one cell (*N* = 1,095). **c**, Left: UMAP plot visualizing CD11c^−^ and CD11c^+^ cells from untreated, LPS-treated and ABX-treated *Cx3cr1*^*CreERT2*^*R26*^*Confetti*^*5×FAD*^*+*^ animals at an early stage of disease relative to the identified cell clusters (C0–C4, dotted outlines) (see color legend, right). Right: stacked bar plot depicting the relative composition of microglial clusters with respect to the cells’ CD11c signal and their treatment group (see color legend). **d**, Heatmap presenting normalized expression of the 20 most differentially expressed genes per cluster. Expression levels are encoded by color as shown in the legend. **e**, Volcano plots with pseudobulk gene expression comparing differential gene expression between PAM and non-PAM (top), between PAM cluster 0 and non-PAM cluster 2 (middle) and between PAM cluster 1 and non-PAM cluster 2 (bottom). The –log_10_-transformed adjusted *P* value (*P* adjusted, *y* axis) is plotted against the log_2_-transformed FC in expression between the indicated cell groups or clusters (*x* axis). Genes under log_2_FC and –log_10_ *P* value cutoff are shown in gray (NS). Genes above log_2_FC but under *P* value cutoff are shown in green, and genes above log_2_FC and –log_10_ *P* value cutoff are shown in red. **f**, GSEA of the pseudobulk differential gene expression in non-PAM versus non-PAM after LPS treatment. HALLMARK_INFLAMMATORY_RESPONSE: normalized enrichment score (NES) = 2, *P* = 0.0, false discover rate (FDR) *q*-value = 0.0. HALLMARK_INTERFERON_GAMMA_RESPONSE: NES = 1.62, *P* = 0.0, FDR *q* = 0.002. HALLMARK_TNFA_SIGNALING_VIA_NFKB: NES = 2.66, *P* = 0.0, FDR *q* = 0.0. Enrichment scores and gene hits are plotted. **g**, GSEA of the pseudobulk differential gene expression in non-PAM compared to non-PAM after ABX treatment. GOBP_REGULATION_OF_INNATE_IMMUNE_RESPONSE: NES = −1.51, *P* = 0.002, FDR *q* = 0.13. GOBP_REGULATION_OF_MACROPHAGE_ACTIVATION: NES = −1.34, *P* = 0.1, FDR *q* = 0.15. Enrichment scores and gene hits are plotted. **h**–**j**, Violin plots depicting the indicated Gene Ontology modules in PAM and non-PAM across treatment groups. The module expression was quantified using the UCell algorithm. The indicated *P* values represent the results of pairwise two-tailed Mann–Whitney *U*-tests. Specific *P* values of statistical tests are indicated. NS, not significant.

**Extended Data Fig. 3:**
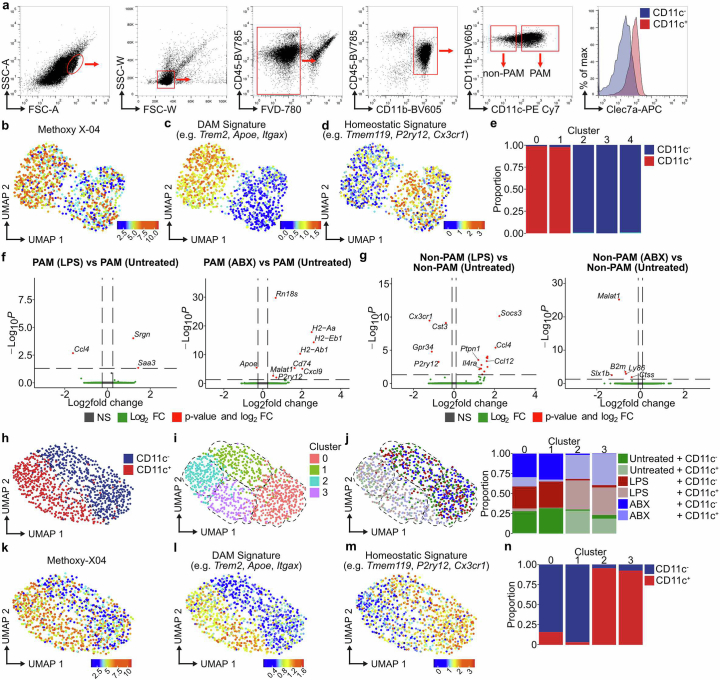
Transcriptional plasticity of non-PAM towards peripheral stimuli and gut dysbiosis is age-dependent. **(a)** Representative FACS sorting strategy. SSC: side scatter, FSC: forward scatter. Histogram for Clec7a expression is shown for CD11c^+^ (red) and CD11c^−^ (blue) microglia. **(b)** UMAP plot depicting Methoxy-X04 levels of index-sorted non-PAM and PAM at ES. Scale refers to fluorescence intensity. Clusters and experimental conditions are consistent with Fig. 4. **(c)** UMAP plot depicting microglia activation signature at ES. The color coding indicates the average expression of DAM core signature minus the average expression of randomly selected background genes. **(d)** UMAP plot depicting microglia homeostatic signature at ES. The color coding indicates the average expression of homeostatic core signature minus the average expression of randomly selected background genes. **(e)** Stacked bar plot of microglial cluster composition (C0-C4) in respect to their CD11c signal: CD11c^−^ (blue) and CD11c^+^ cells (red). **(f-g)** Volcano plots of pseudo bulk gene expression comparing differential gene expression between PAM (**f**) or non-PAM (**g**) are shown. Left: LPS-treated vs untreated, and right: ABX-treated vs untreated at LS are shown. y-axis: –Log10-transformed adjusted P value (P adjusted), x-axis: Log2-transformed fold change (FC). Genes under Log2 FC and –Log10 P value cut off (grey (NS)) are shown. Genes above Log2 FC but under P value cut off (green) and above Log2 FC and –Log10 P value cut off (red) are shown. **(h)** UMAP plot of CD11c^−^ (blue) and CD11c^+^ (red) microglia from untreated, LPS- and ABX-treated *Cx3cr1*^*CreERT2*^*R26*^*Confetti*^*5xFAD*^*+*^ animals at LS. Each symbol represents one cell (N = 1,017 cells). **(i)** UMAP plot of cell clusters (C0-C3) identified from untreated, LPS- and ABX-treated *Cx3cr1*^*CreERT2*^*R26*^*Confetti*^ *5xFAD*^*+*^ animals at LS. Each color represents one distinct cell cluster. Each cluster is outlined by dotted lines. Each symbol represents one cell (N = 1,017 cells). **(j)** Left: UMAP plot of CD11c^−^ and CD11c^+^ cells from untreated, LPS- and ABX-treated *Cx3cr1*^*CreERT2*^*R26*^*Confetti*^*5xFAD*^*+*^ animals at LS relative to the identified cell clusters (C0-C3, dotted outlines) (see color legend). Right: Stacked bar plot of microglial cluster composition in respect to the cells’ CD11c signal and their treatment group (see color legend). **(k)** UMAP plot depicting Methoxy-X04 levels of index-sorted non-PAM and PAM at LS. Scale refers to fluorescence intensity. Clusters and experimental conditions are consistent with Fig. 4. **(l)** UMAP plot depicting microglia activation signature at LS. The color coding indicates the average expression of DAM core signature minus the average expression of randomly selected background genes. **(m)** UMAP plot depicting microglia homeostatic at LS. The color coding indicates the average expression of homeostatic core signature minus the average expression of randomly selected background genes. **(n)** Stacked bar plot depicting microglial cluster composition (C0-C3) at LS in respect to their CD11c signal: CD11c^−^ (blue) and CD11c^+^ cells (red).

To elucidate which molecular events orchestrate clonal expansion of PAM from adjacent non-PAM, we examined the introduced transcriptional changes and heterogeneity in both compartments by environmental factors. First, we profiled PAM and non-PAM from all treatment groups at the early stage by scRNA-seq (Extended Data Fig. 3a). For single-cell sorting, non-PAM were defined by CD45^+/low^CD11b^+^CD11c^−^ and PAM by CD45^+/low^CD11b^+^CD11c^+^ expression. Separation of non-PAM and PAM by CD11c expression was further confirmed by distinct expression levels of Clec7a, which was elevated in amyloid-adjacent PAM, as previously described^11^. Subsequent transcriptional profiling of individual cells on a uniform manifold approximation and projection (UMAP) plot segregated CD11c^+^ PAM and CD11c^−^ non-PAM (Fig. 4a). Furthermore, index-sorted PAM showed enhanced fluorescence intensity for Methoxy-X04 compared to non-PAM (Extended Data Fig. 3b). Unsupervised clustering subdivided all annotated microglia to five clusters (C0–C4) (Fig. 4b), with C0 and C1 enriched in PAM and C2–C4 enriched in non-PAM (Fig. 4b). Microglial activation signature was enriched in PAM clusters (C0 and C1), whereas the non-PAM clusters C2–C4 expressed a homeostatic microglial signature (Fig. 4b and Extended Data Fig. 3c–e). Upon LPS and ABX treatment, we observed prominent transcriptional changes in non-PAM enriched clusters from untreated, LPS-treated and ABX-treated mice, respectively (Fig. 4c). Notably, non-PAM exhibited a clear enrichment of cells in distinct clusters after ABX (C3) and LPS (C4) treatment, which were transcriptionally distinct from the untreated non-PAM cluster C2 (Fig. 4c,d). In contrast, no treatment-associated microglial clusters were found in PAM. To elucidate potential effects of the treatments, we compared gene expression changes between the identified clusters. Differentially expressed genes between the clusters pointed to the enrichment of genes such as *Cst7*, *Apoe*, *Axl*, *Igf1* or *Ctsd* in C0 and C1, mainly consisting of PAM, compared to the other clusters C2–C4, mainly consisting of non-PAM (Fig. 4d,e).

By using gene set enrichment analysis (GSEA) to decipher alterations induced in the non-PAM cell states upon ABX or LPS treatment, we revealed a profound induction of gene sets associated with inflammation, including interferon gamma (IFNγ) signaling and nuclear factor kappa-light-chain-enhancer of activated B cells (NF-κB) activation via tumor necrosis factor (TNF) signaling, in the non-PAM upon LPS treatment (Fig. 4f). In contrast, ABX treatment induced a downregulation of pro-inflammatory gene sets, including genes associated with regulation of innate immune response or regulation of macrophage activation (Fig. 4g). Next, we tested whether the transcriptional unresponsiveness of PAM is due to cellular exhaustion and senescence (Fig. 4h–j). Genes related to the MyD88-dependent pathway, as well as to senescence, were not altered in PAM compared to non-PAM, whereas the MyD88-independent signaling pathway was slightly changed. As expected, TREM2-dependent and TREM2-independent pathways were found to be differentially expressed by PAM and non-PAM (Fig. 4i). Notably, when we analyzed adaptation of cellular states after ABX and LPS treatment in late stage, only minor transcriptional changes were detectable, with no treatment-associated clustering in PAM and non-PAM (Extended Data Fig. 3f–n)

Taken together, using scRNA-seq, we describe distinct age-dependent and context-dependent microglial states within non-PAM but not in PAM. Notably, these findings suggest that non-PAM comprise more responsive and modular microglia states, which could represent potential targets during amyloid pathology.

### Chromatin accessibility patterns separate non-PAM from PAM

**Extended Data Fig. 4:**
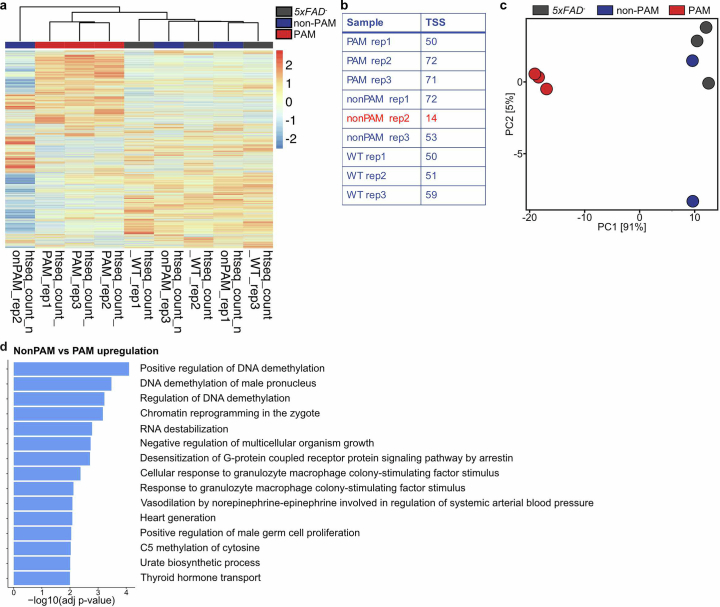
Quality control of chromatin accessibility data and gene ontology analysis. **(a)** Heat map displaying chromatin accessibility in consensus ATAC-Seq peak set (59,293 total peaks) across all samples. Each column of the heat map corresponds to an individual sample. Each row corresponds to a region of the consensus peak list. Normalized counts (DESeq2) from each sample are scaled per region. **(b)** Transcriptional start site enrichment (TSSe) scores for all ATAC-seq datasets. Datasets with TSSe > 15 were used for downstream analysis. Non-PAM rep2 was removed before downstream analysis. **(c)** Principal component analysis (PCA) of chromatin accessibility profiles in microglia from 5xFAD- (black) or 5xFAD+ mice (non-PAM: blue and PAM: red). Each symbol represents microglia isolated from one mouse. **(d)** Significantly enriched pathways of genes linked to DARs with increased chromatin accessibility in non-PAM compared to PAM.

To define the different transcriptional responsiveness of non-PAM and PAM on the molecular level, we next performed assay for transposase-accessible chromatin using sequencing (ATAC–seq) to profile chromatin accessibility in microglia from *5×FAD*^*−*^ or PAM and non-PAM from *5×FAD*^*+*^ at early stage of the disease. After quality control, we excluded one sample (non-PAM rep 2) owing to low signal-to-noise ratio compared to the other samples (Extended Data Fig. 4a,b).

**Fig. 5:**
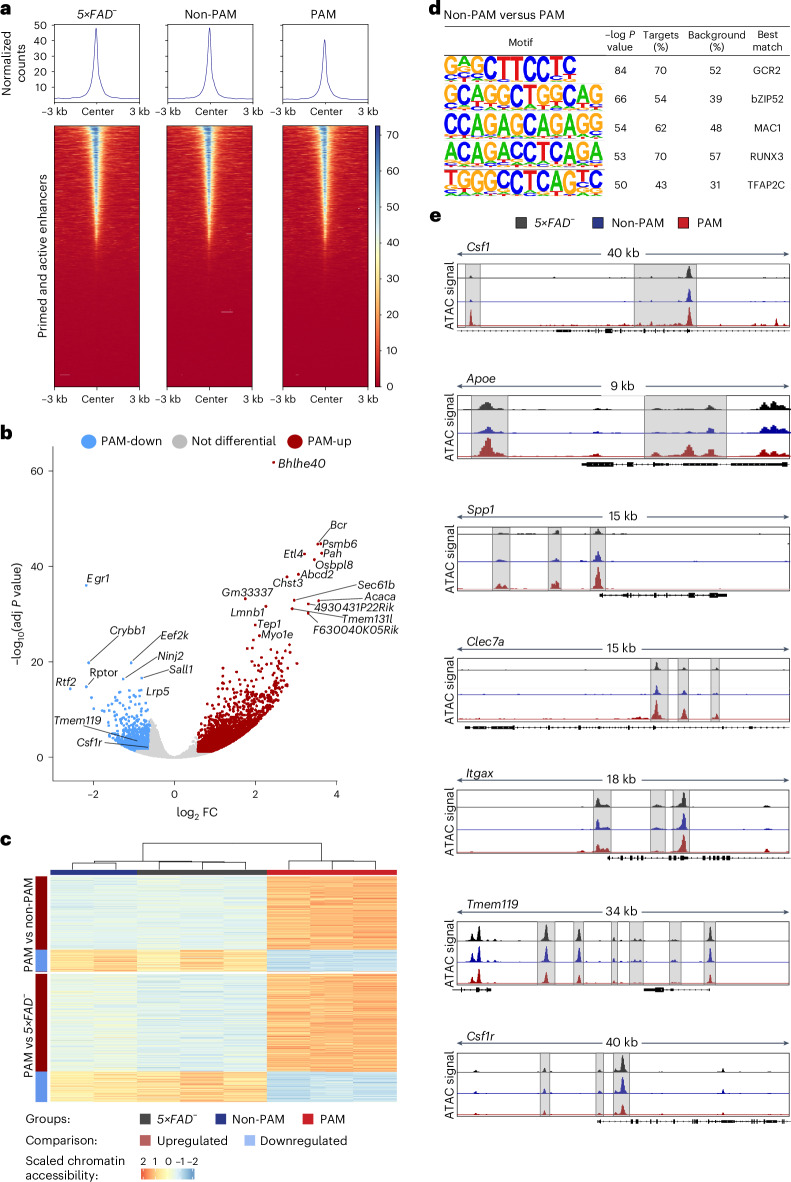
Distinct chromatin accessibility landscapes in microglial subsets depending on their spatial relationship to pathology. **a**, Heatmaps of chromatin accessibility in microglia from *5×FAD*^*−*^ or *5×FAD*^*+*^ mice (non-PAM and PAM) at 22,929 previously defined primed and active microglial enhancer regions. **b**, Volcano plot of chromatin accessibility differences of peaks between PAM and non-PAM. In total, 4,360 peaks were more accessible in PAM (red; PAM-up), and 1,301 showed higher accessibility in non-PAM (blue; PAM-down). DARs (FDR < 0.05, log_2_FC ≥ 0.584 or log_2_FC ≤ −0.584) were associated with nearby genes using ChIPSeeker. Labeling on the plot refers to predicted target genes. For DAR to gene annotations, see Supplementary Table 1. **c**, Heatmap depicting chromatin accessibility for individual samples at DARs. The upper part of the heatmap shows DARs of PAM versus non-PAM; the lower part shows DARs of PAM versus *5×FAD*^*−*^. Comparison between PAM and non-PAM revealed 5,661 DARs with 4,360 regions showing a higher accessibility and 1,301 regions with decreased accessibility. In the comparison of PAM versus *5×FAD*^−^, out of a total of 7,637 DARs, 5,848 showed increased accessibility, and 1,789 showed decreased accessibility (FDR < 0.05, log_2_FC ≥ 0.584 or log_2_FC ≤ −0.584). **d**, Transcription factor binding motifs enriched in DARs with higher accessibility in non-PAM compared to PAM. **e**, Integrative Genome Viewer tracks displaying normalized profiles of *Csf1*, *Apoe*, *Spp1*, *Clec7a*, *Itgax*, *Tmem119* and *Csf1r* loci in microglia from *5×FAD*^*−*^ mice (black) or *5×FAD*^*+*^ mice (non-PAM: blue; PAM: red). For display, DARs are highlighted, and data from all replicates were merged (*n* = 3 for *5×FAD*^−^ and PAM, *n* = 2 for non-PAM).

PAM and non-PAM from *5×FAD*^*+*^ mice and microglia from *5×FAD*^*−*^ mice displayed similar chromatin accessibility profiles at previously defined primed and active enhancer sites (Fig. 5a). Furthermore, principal component analysis (PCA) demonstrated that non-PAM and microglia from non-transgenic mice clustered together and separated from PAM (Extended Data Fig. 4c). Next, we identified differentially accessible regions (DARs) and found 4,360 regions with higher accessibility (PAM-up) and 1,301 regions with lower accessibility (PAM-down) in PAM compared to non-PAM (Fig. 5b,c and Supplementary Table 1). Chromatin accessibility levels at DARs were similar between non-PAM and microglia from *5×FAD*^*−*^ mice (Fig. 5c). PAM-down regions were associated with genes such as *Egr1*, *Crybb1* and *Nunj2* but also homeostatic microglia genes such as *Tmem119* and *Csf1r*. PAM-up regions were associated with genes involved in cellular stress response, such as *Bcr*, *Psmb6*, *Pah* or *Tep1* (Fig. 5b). Moreover, we found binding motifs for the glycolytic transcriptional activator GRC2, the copper-dependent transcription factor MAC1 or the proliferation-arresting transcription factor RUNX3 enriched in peaks with higher accessibility in non-PAM (Fig. 5d). Chromatin accessibility in PAM was increased at DARs assigned to DAM signature genes, such as *Csf1*, *Apoe*, *Spp1*, *Clec7a* and *Itgax* (Fig. 5e). On the other hand, non-PAM and microglia from *5×FAD*^*−*^ mice showed higher chromatin accessibility at DARs associated with homeostatic microglia signature genes such as *Tmem119* but also *Csf1r* (Fig. 5e). Regions with higher accessibility in non-PAM were furthermore associated with genes involved in DNA methylation and chromatin reprogramming (Extended Data Fig. 4d). Collectively, these observations substantiate the conclusion that PAM adapt distinct chromatin accessibility profiles at the site of amyloid plaques, whereas non-PAM are more similar to the chromatin accessibility landscape of homeostatic microglia.

### Csf1 restricts PAM generation and improves amyloid pathology

Given the profound differences in the transcriptome and accessible chromatin landscapes between non-PAM and PAM, we next examined whether these two microglia populations can be targeted distinctly. Because we identified non-PAM as a dynamic and modular microglia population during early stages of AD, we hypothesized that this spatially distinct microglial state might offer a plausible candidate population to test focused therapeutic interventions. In both the scRNA-seq data (Fig. 4e) and the ATAC–seq analysis (Fig. 5e), we revealed higher transcript levels and an open chromatin region for *Csf1r* in non-PAM compared to PAM, making CSF1R a potential molecular target in this population.

**Fig. 6:**
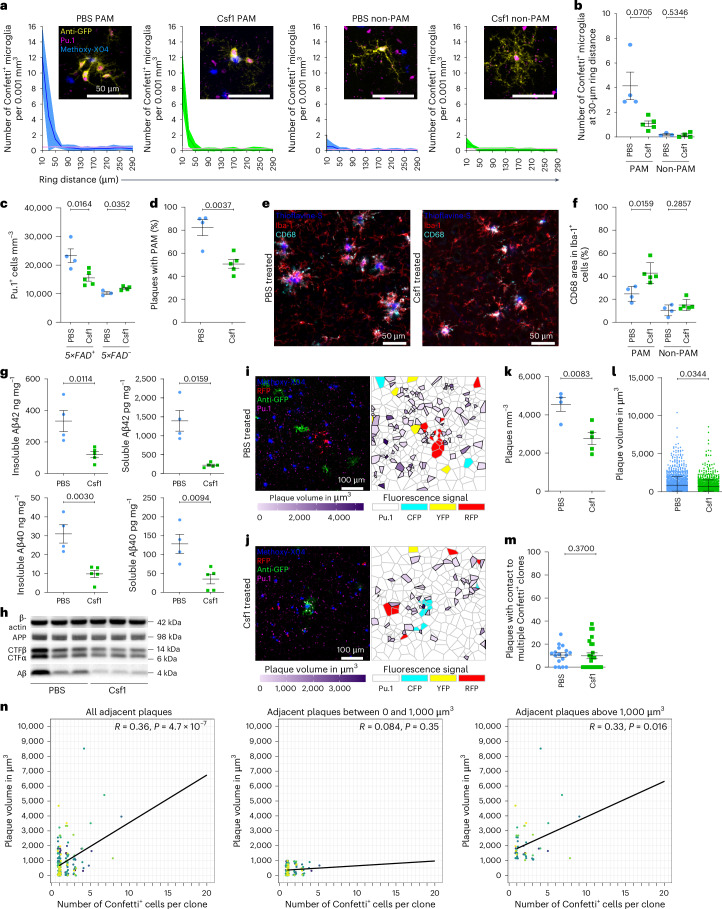
Engagement of Csf1r by Csf1 mitigates clonal expansion of PAM, elevates phagocytic capacity of PAM and attenuates amyloid pathology at early stage of disease. **a**, MC simulation: densities of Confetti^+^ PAM and non-PAM in *Cx3cr1*^*CreERT2*^*R26*^*Confetti*^*5×FAD*^*+*^ animals after treatment with Csf1 (green) (*n* = 5) or PBS (blue) (*n* = 4) at early stage of disease, relative to randomized datasets (pink). Densities are displayed relative to measured distances (that is, ring distance (µm)). Means per group and 98% confidence intervals are shown. Insets: representative images of anti-GFP-labeled Confetti^+^ (yellow) Pu.1^+^ (magenta) microglia in relation to Methoxy-X04 (blue). Scale bars, 50 µm. **b**, Quantification of Confetti^+^ cells from *Cx3cr1*^*CreERT2*^*R26*^*Confetti*^*5×FAD*^*+*^ animals after treatment with Csf1 (green) (*n* = 5) or PBS (blue) (*n* = 4). Each symbol represents one animal. Mean ± s.e.m. is shown. Specific *P* values of statistical tests are indicated. **c**, Quantification of Pu.1^+^ cells per mm^3^ in *Cx3cr1*^*CreERT2*^*R26*^*Confetti*^*5×FAD*^*+*^ animals and littermate controls after treatment with Csf1 (green) (*n* = 5) or PBS (blue) (*n* = 4). Each symbol represents one animal. Mean ± s.e.m. is shown. Specific *P* values of statistical tests are indicated. **d**, Quantification of plaques with PAM in *Cx3cr1*^*CreERT2*^*R26*^*Confetti*^*5×FAD*^*+*^ animals after treatment with Csf1 (green) (*n* = 5) or PBS (blue) (*n* = 4). Each symbol represents one animal. Mean ± s.e.m. is shown. Specific *P* values of statistical tests are indicated. **e**, Representative images of CD68^+^Iba-1^+^ microglia from *Cx3cr1*^*CreERT2*^*R26*^*Confetti*^*5×FAD*^*+*^ mice after treatment with PBS (left) or Csf1 (right). Immunofluorescence is shown for CD68 (cyan), Iba-1 (red) and thioflavine-S (blue). Scale bars, 50 µm. **f**, Quantification of CD68^+^Iba-1^+^ PAM and non-PAM in *Cx3cr1*^*CreERT2*^*R26*^*Confetti*^*5×FAD*^*+*^ animals after treatment with Csf1 (green) (*n* = 5) or PBS (blue) (*n* = 4). Each symbol represents one animal. Mean ± s.e.m. is shown. Specific *P* values of statistical tests are indicated. **g**, ELISA of human Aβ_1–40_ and Aβ_1–42_ peptides in insoluble fractions (left) and soluble fractions (right) of *Cx3cr1*^*CreERT2*^*R26*^*Confetti*^*5×FAD*^*+*^ animals after treatment with Csf1 (green) (*n* = 5) or PBS (blue) (*n* = 4). Each symbol represents one animal. Mean ± s.e.m. is shown. Specific *P* values of statistical tests are indicated. **h**, Representative western blots for β-actin, APP, CTFβ, CTFα and human Aβ in brain lysates from the frontal cortices from *Cx3cr1*^*CreER*^*R26R*^*Confetti*^*5×FAD*^*+*^ mice treated with CSF1 or PBS, respectively. Each lane represents one animal, *n* = 3 per group. **i**, Left: representative image from a *Cx3cr1*^*CreERT2*^*R26*^*Confetti*^*5×FAD*^*+*^ animal treated with PBS. Methoxy-X04 (blue), anti-RFP (red), anti-GFP (green) and Pu.1 (magenta) are shown. Scale bar, 100 µm. Right: Voronoi grid thereof visualizing plaque sizes and the positioning of Confetti^−^Pu.1^+^ and Confetti^+^Pu.1^+^ microglia. Confetti^−^Pu.1^+^ (white), CFP^+^Pu.1^+^ (cyan), YFP^+^ Pu.1^+^ (yellow) and RFP^+^Pu.1^+^ (red) microglia are shown. **j**, Left: representative image from a *Cx3cr1*^*CreERT2*^*R26*^*Confetti*^*5×FAD*^*+*^ animal treated with Csf1. Methoxy-X04 (blue), anti-RFP (red), anti-GFP (green) and Pu.1 (magenta) are shown. Scale bar, 100 µm. Right: Voronoi grid thereof visualizing plaque sizes and the positioning of Confetti^−^Pu.1^+^ and Confetti^+^Pu.1^+^ microglia. Confetti^−^Pu.1^+^ (white), CFP^+^Pu.1^+^ (cyan), YFP^+^Pu.1^+^ (yellow) and RFP^+^Pu.1^+^ (red) microglia are shown. **k**, Quantification of number of plaques in *Cx3cr1*^*CreERT2*^*R26*^*Confetti*^*5×FAD*^*+*^ mice after treatment with Csf1 (green) (*n* = 5) or PBS (blue) (*n* = 4). Each symbol represents one animal. Mean ± s.e.m. is shown. Specific *P* values of statistical tests are indicated. **l**, Quantification of individual plaque sizes after treatment with Csf1 (green) (*n* = 5) or PBS (blue) (*n* = 4). Each symbol represents one analyzed plaque (*N* = 1,016–1,346 plaques per group). Median ± interquartile range is shown. Specific *P* values of statistical tests are indicated. **m**, Quantification of plaques associated with multiple Confetti^+^ PAM clones in frontal cortices of *Cx3cr1*^*CreERT2*^*R26*^*Confetti*^*5×FAD*^*+*^ animals after treatment with Csf1 (green) (*n* = 5, *N* = 25) or PBS (blue) (*n* = 4, *N* = 17). Each symbol represents individual analyzed images. Mean ± s.e.m. is shown. Specific *P* values of statistical tests are indicated. **n**, Correlations between Confetti^+^ microglia clone size and adjacent Methoxy-X04^+^ plaques after Csf1 treatement. Colors of individual points represent separately analyzed images (*N* = 25) across individuals (*n* = 5). Black line indicates linear regression. Left: correlation for all Methoxy-X04^+^ plaque sizes (R = 0.36, ***P *=* 4.7 × 10^−7^). Middle: correlation for <1,000-µm^3^-sized Methoxy-X04^+^ plaques (*R* = −0.084, NS *P* *=* 0.35). Right: correlation for Methoxy-X04^+^ plaques >1,000 µm^3^ (*R* = 0.33, *P *=* 0.016) are shown. Source data

**Extended Data Fig. 5:**
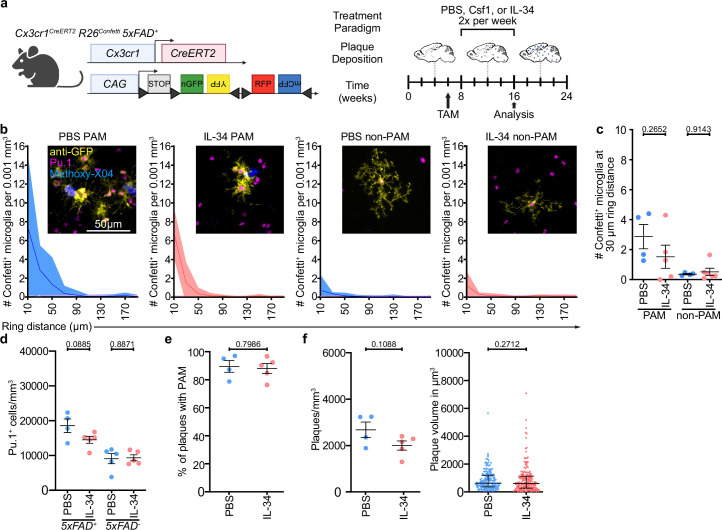
IL-34 treatment does not affect clonal expansion of PAM or amyloid pathology. **(a)** Experimental scheme for Csf1 and IL-34 treatment of *Cx3cr1*^*CreERT2*^*R26*^*Confetti*^*5xFAD*^*+*^ mice. **(b)** Monte Carlo simulation: Densities of Confetti^+^ PAM and non-PAM in *Cx3cr1*^*CreERT2*^*R26*^*Confetti*^*5xFAD*^*+*^ animals after treatment with IL-34 (red) (n = 5) or PBS (blue) (n= 4) at early stage of disease, relative to randomized datasets (pink). Densities are displayed relative to measured distances (that is ring distance; µm) between Confetti^+^ cells. Means per group and 98 % confidence intervals are shown for experimental datasets and randomized datasets. Representative confocal images of anti-GFP labeled Confetti^+^ (yellow) Pu.1^+^ (magenta) microglia in relation to Methoxy-X04 (amyloid beta, blue) are shown for each group. Scale bar = 50 µm. **(c)** Quantification of Confetti^+^ cells from *Cx3cr1*^*CreERT2*^*R26*^*Confetti*^*5xFAD*^*+*^ animals after treatment with IL-34 (red) (n = 5) or PBS (blue) (n= 4) at early stage of disease. Each symbol represents one animal. Specific *P* values of statistical tests are indicated. **(d)** Quantification of Pu.1^+^ cells per mm^3^ in *Cx3cr1*^*CreERT2*^*R26*^*Confetti*^*5xFAD*^*+*^ animals and littermate controls after treatment with IL-34 (red) (n = 5) or PBS (blue) (n= 4) at early stage of disease. Each symbol represents one animal. Mean ± s.e.m. is shown. Specific *P* values of statistical tests are indicated. **(e)** Quantification of the percentage of plaques with associated PAM in *Cx3cr1*^*CreERT2*^*R26*^*Confetti*^*5xFAD*^*+*^ animals after treatment with IL-34 (red) (n = 5) or PBS (blue) (n= 4) at early stage of disease. Each symbol represents one animal. Mean ± s.e.m. is shown. Specific *P* values of statistical tests are indicated. **(f)** Quantification of number of plaques per mm^3^ (left) and quantification of individual plaque sizes (right) in the frontal cortices of *Cx3cr1*^*CreERT2*^*R26*^*Confetti*^*5xFAD*^*+*^ mice after treatment with IL-34 (red) (n = 5, N = 555) or PBS (blue) (n= 4, N = 547) at early stage of disease. Left: Each symbol represents one animal. Mean ± s.e.m. is shown. Right: Each symbol represents one analyzed plaque. Median ± interquartile range is shown. Specific *P* values of statistical tests are indicated. Source data

To this end, we peripherally injected *Cx3cr1*^*CreERT2*^*R26*^*Confetti*^*5×FAD*^*+*^ mice and controls with the CSF1R ligands Csf1 or interleukin (IL)-34 at early stages of amyloid pathology (Extended Data Fig. 5a). After Csf1 treatment, clonal expansion of Confetti^+^Pu.1^+^ PAM was diminished (Fig. 6a,b), but no alterations in the expansion of non-PAM were induced. Overall microglial cell number was reduced after Csf1 treatment in comparison to PBS-injected *Cx3cr1*^*CreERT2*^*R26*^*Confetti*^*5×FAD*^*+*^ mice (Fig. 6c) but slightly elevated in Csf1-treated *Cx3cr1*^*CreERT2*^*R26*^*Confetti*^*5×FAD*^*−*^ mice (Fig. 6c). Furthermore, fewer amyloid plaques were associated with CD11c^+^ PAM (Fig. 6d). In contrast to non-PAM, PAM showed an enlarged CD68^+^ lysosomal compartment, suggesting increased microglial phagocytosis at amyloid deposits (Fig. 6e,f). Analysis of soluble and insoluble Aβ_1–40_ and Aβ_1–42_ levels revealed a strong reduction upon Csf1 treatment (Fig. 6g), whereas amyloid precursor protein (APP) processing itself was not affected (Fig. 6h). To gain further insights into the seeding and distribution of amyloid deposits and their relation to Confetti^+^Pu.1^+^ PAM clones, we performed a semi-automated analysis with Voronoi gridding after Csf1 treatment compared to PBS-injected animals (Fig. 6i,j). We observed reduced numbers and volumes of amyloid plaques after Csf1 application (Fig. 6k,l). Moreover, we found no change in the amount of plaques with contact to multiple Confetti^+^ clones (Fig. 6m). Csf1 not only reduced amyloid plaque volumes and PAM clone sizes but also induced a higher correlation between these parameters (Fig. 6n). Large amyloid deposits (>1,000 µm^3^), which outcompeted PAM clones before, became now highly correlative with PAM clone size, indicating that Csf1 treatment modulated the expansion of PAM, which restricted amyloid growth (Fig. 6m). In contrast to Csf1, the other CSF1R ligand, IL-34, did not influence clonal expansion of Confetti-labeled PAM or non-PAM compared to PBS-treated controls (Extended Data Fig. 5b,c). Similarly, IL-34 treatment did not affect the microglial cell number nor the decoration of amyloid plaques with PAM (Extended Data Fig. 5d,e). Furthermore, plaque numbers and volumes were not altered upon IL-34 treatment (Extended Data Fig. 5f).

In sum, Csf1 treatment, but not IL-34 application, enhances phagocytic capacity of PAM and ameliorates PAM expansion at early stages of disease, thereby facilitating restriction of amyloid pathology. Our data further suggest that these effects subsequently modulate the pathology-driven dynamics between microglia states, targeting the transition of non-PAM to PAM by engagement with CSF1R, resulting in diminished disease progression.

### Csf1 shapes amyloid-competent PAM from non-PAM

**Fig. 7:**
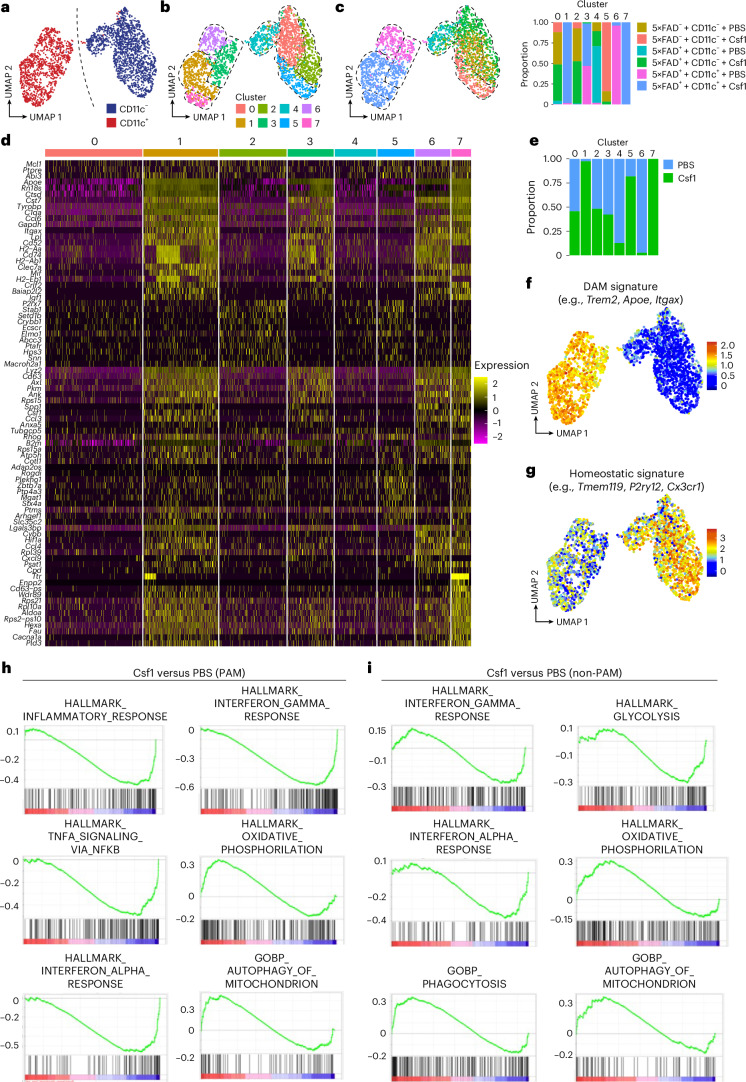
Csf1 treatment beneficially modulates functional and metabolic features of non-PAM-derived PAM, making these cells competent to restrict amyloid pathology at early stages of neurodegeneration. **a**, UMAP plot demonstrating the distribution of analyzed CD11c^−^ (blue) and CD11c^+^ (red) microglia from PBS-treated or Csf1-treated *Cx3cr1*^*CreERT2*^*R26*^*Confetti*^*5×FAD*^*+*^ animals and controls at an early stage of disease. Each dot represents one cell (*N* = 2,687). **b**, UMAP visualizing the distribution of cell clusters (C0–C7) identified by unsupervised clustering of all analyzed cells from PBS-treated or Csf1-treated *Cx3cr1*^*CreERT2*^*R26*^*Confetti*^*5×FAD*^*+*^ animals and littermate controls at an early stage of disease. Each color represents one distinct cell cluster. Each cluster from **b** is highlighted by dotted lines. **c**, Left: UMAP representation displaying the distribution of clusters according to the treatment regimens (PBS-treated or Csf1-treated *Cx3cr1*^*CreERT2*^*R26*^*Confetti*^ *5×FAD*^*+*^ animals and littermates controls at an early stage). Cell clusters from **b** (C0–C7) are displayed by dotted lines. Right: stacked bar blot depicting the relative composition of microglial clusters (C0–C7) with respect to their treatment group. **d**, Heatmap presenting log_2_FC of the 20 most differentially expressed genes per cluster. Expression levels are encoded by color as shown in the color legend. **e**, Stacked bar plot depicting the relative composition of microglial clusters (C0–C7) with respect to their treatments. **f**, UMAP feature plot depicting the expression of genes associated with microglial activation (*Apoe*, *Axl*, *Bhlhe40*, *Clec7a*, *Csf1*, *Cst7*, *Ctsb*, *Ctsd*, *Ctsl*, *Cybb*, *Fabp5*, *Fth1*, *Itgax*, *Gnas*, *Gpnmb*, *Grn*, *Il1b*, *Lgals3*, *Lilrb4*, *Lpl*, *Lyz2*, *Msr1*, *Nos2*, *Spp1*, *Tfec*, *Trem2*, *Tyrobp* and *Vegfa*). The color scale reflects the relative enrichment of the gene set expression per cell, as calculated by the AddModuleScore function. **g**, UMAP representing the expression of genes associated with a homeostatic microglia signature *(P2ry12*, *Csf1r*, *Cx3cr1*, *Tmem119*, *Pu.1* and *Sall1*). The color scale reflects the relative enrichment of the gene set expression per cell, as calculated by the AddModuleScore function. **h**, GSEA of all PAM after Csf1 compared to PBS treatment. Plots for the running sum of S are shown for defined gene sets together with the maximum enrichment score (ES). **i**, GSEA of all non-PAM after Csf1 treatment. Plots for the running sum of S are shown for defined gene sets together with the maximum ES.

To test whether and how Csf1 treatment modulates pathology-associated microglia states and the transition of non-PAM to PAM, we performed scRNA-seq of both after 4 weeks of Csf1 or PBS treatment in *Cx3cr1*^*CreERT2*^*R26*^*Confetti*^*5×FAD*^*+*^ mice and controls at early stage of the disease. This early timepoint was chosen to obtain a sufficient number of PAM, in case longer Csf1 treatment would completely block the development of PAM around plaques. Transcriptomic profiling showed that non-PAM and PAM clearly segregated on a UMAP plot (Fig. 7a). Eight distinct microglial clusters, designated C0–C7, were identified when performing unsupervised clustering (Fig. 7b–d). We next tested the distribution of analyzed PAM and non-PAM in the different treatment groups across the clusters (Fig. 7c,e). Clusters C1, C3, C6 and C7 were enriched in PAM, whereas C0, C2, C4 and C5 mainly included non-PAM (Fig. 7c,e), as indicated by the expression of disease-associated signature genes in C1, C3, C6 and C7 and homeostasis signature genes in C0, C2, C4 and C5 (Fig. 7d,f,g). We identified two specific Csf1-induced clusters in the CD11c^+^ PAM population, namely C1 and C7, but no cluster specifically enriched for CD11c^−^ non-PAM. Only one Csf1-induced cluster (C5) within the CD11c^−^ microglia was observed in *Cx3cr1*^*CreERT2*^*R26*^*Confetti*^*5×FAD*^*−*^ controls. Notably, both PAM and non-PAM retained their respective homeostatic (*Tmem119*, *P2ry12*, *Cx3cr1*, etc.) or activated (*Trem2*, *Apoe*, *Itgax*, etc.) gene panel upon Csf1 treatment, indicating that there is no therapy-induced shift of non-PAM toward PAM involving their core signatures (Fig. 7f,g).

To better understand the beneficial effects of Csf1 on microglial dynamics and disease progression during amyloid pathology, we performed GSEA on pseudobulk gene expression analysis of PAM isolated from Csf1-treated or PBS-treated *Cx3cr1*^*CreERT2*^*R26*^*Confetti*^*5×FAD*^*+*^ mice (Fig. 7h). Here, Csf1 treatment induced a profound decrease in gene sets associated with inflammatory response, including type I or type II interferon signaling or NF-κB activation via TNF signaling (Fig. 7h), and an upregulation of genes associated with metabolic regulation toward autophagy and oxidative phosphorylation (Fig. 7h). Notably, similar trends were observed in non-PAM after Csf1 treatment (Fig. 7i) indicating that both spatially distinct microglial populations are highly modulated by Csf1, even though *Csf1r* expression was found to be downregulated in PAM. This highly suggests cell-autonomous effects of Csf1 on non-PAM directly and modulation of their differentiation to more amyloid-competent PAM. Here, Csf1 beneficially affects these dynamic microglia states during their transition phase before PAM clones are orchestrated at the plaque site.

Taken together, our data indicate that the beneficial effect of Csf1 treatment does not per se change the core signatures of non-PAM and PAM but, rather, alleviates pathways of inflammation and increases mitochondrial features, suggesting an improvement of the functional fitness (for example, phagocytosis and metabolism) and the generation of an amyloid-competent and restrictive PAM population.

## Discussion

In this study, a combination of different multicolor fate-mapping mouse models along with transcriptomic and epigenomic profiling revealed defined spatial and temporal dynamics between PAM and non-PAM during amyloid pathology in female mice. We identified non-PAM as more transcriptionally responsive to external stimuli and therapeutic modulations, such as engagement of CSF1R by Csf1 treatment. This treatment paradigm efficiently modulates differentiation of non-PAM to amyloid-restrictive PAM with subsequent amelioration of amyloid pathology.

Our analyses found dynamic microglial turnover between non-PAM and PAM, with selective clonal expansion of PAM originating from adjacent individual non-PAM. Non-PAM-specific Confetti labeling allowed us to determine that the PAM compartment is maintained by continuous input of cells derived from individual neighboring non-PAM. The recruitment and accumulation of microglia to the plaque sites were reported previously using a fate-mapping model with single-colored microglia to examine microglial kinetics at amyloid deposition^2^. However, this study did not further explore the dynamics and function of non-PAM during amyloid pathology progression.

The generation of PAM has been debated as a driving force of disease progression in AD^18,19^ but also as playing a beneficial role in controlling amyloid pathology progression^25,26^. However, the functional role of non-PAM in this process and its potential contribution to the disease pathology as the origin of PAM clones has been largely neglected. Microglia associated with extracellular amyloid, possessing a distinct gene expression profile also known as DAM^11^ or MgnD^17^, were thus far considered as the only pathophysiologically relevant microglial population in neurodegeneration^27^. Recent studies targeting PAM-specific molecules and signaling pathways, including TREM2 and APOE, revealed differing results on the disease progression and outcome^18–20,28^.

To perform marker-based discrimination between PAM and non-PAM, we found CD11c as a marker for PAM and Tmem119 to be highly restricted to non-PAM. In contrast, other markers tested showed less specificity, including Clec7a, which was recently reported to be highly specific for DAM in demyelination and proliferative microglia during development^29^. In line with our findings, this shows that microglia can share certain disease-associated microglia signature genes, but that there are subtle differences between microglia states across diseases, which need to be carefully addressed in the future.

Taking the relative longevity of microglia into consideration^22,30^, our findings of two spatially distinct but interconnected microglial cell populations during neurodegeneration are of potentially considerable therapeutic relevance. Our data further highlight that, within PAM and non-PAM, distinct transcriptional microglia states exist that might be connected to dedicated functions in future studies. The transition of non-PAM toward PAM might also represent a transition through distinct transcriptional states^31^. Therefore, subset-specific targeting is highly desirable in a disease setting to ensure that the homeostatic functions of microglia in non-affected brain regions are maintained, as seen, for example, after microglia depletion by CSF1R inhibition^32,33^. However, only limited information is currently available on the specific lifetime of PAM and non-PAM during amyloid pathology and the dynamics between these two. The proliferative transition of non-PAM to clonally expanding PAM described here is supported by recent studies demonstrating that prolonged and enhanced proliferation of microglia due to amyloid deposition results in a replicative senescence in microglia and the development of senescent DAM^34,35^.

Our findings on the effects of peripheral stimuli on clonal expansion of PAM are further in line with studies in patients showing that chronic inflammation is strongly associated with an increased risk of developing AD^36^. Similarly, studies in transgenic AD mouse models revealed that a single dose or short-term application of LPS alone induced an increase in plaque load and a decrease in Aβ uptake^24,36,37^. However, the effects on both microglia populations, with enhanced clonal expansion of PAM and priming of non-PAM, were not described in previous studies. Thus, a single injection of LPS might induce different alterations compared to a chronic administration of LPS, with the latter being a better model to study chronic inflammation in humans and its effects on AD.

Previous studies reported reduced amyloid pathology and differential effects on microglia by antibiotic-induced depletion of microbiota^23,38,39^. Most likely, the differences between our study and other reported findings are due to different transgenic lines, sexes, ages and brain regions analyzed. Although we found that depletion of endogenous microbiota mostly affects the non-PAM population and clonal PAM expansion at early stage of disease, our data support the assumption that peripheral stimuli such as LPS or depletion of endogenous microbiota affect microglia dynamics and clonality in an age-dependent manner during pathology.

One important characteristic of non-PAM is the higher gene expression and accessible gene locus for *Csf1r*, which were both clearly reduced in the PAM. This downregulation of *Csf1r* gene expression was previously reported for DAM and MgnD microglia states^11,17^. CSF1R is an essential differentiation and survival factor for microglia^6^. The two known ligands for CSF1R are IL-34 and Csf1, which are derived from different cellular sources in the central nervous system^40–42^. Regulation of both ligands was reported in the brains of patients with AD: whereas *CSF1* gene expression was increased in hippocampal regions of patients with AD, *IL34* expression was downregulated^43^.

The therapeutic potential of modulating CSF1R signaling in brain diseases has been proposed on several levels. CSF1R inhibition or depletion was achieved before by genetic modifications or pharmacological inhibition in models of AD^44–46^. Of note, one recent study suggested that pharmacological CSF1R inhibition can be used to potentially modulate development of PAM in AD and improve amyloid deposition by blocking senescent proliferation^35^. However, depleting effects on other macrophage populations and microglia in unaffected brain regions after CSF1R inhibition can hamper its potential therapeutic use^32^. Furthermore, genetic mutations reducing CSF1R signaling capacity are associated with development of hereditary diffuse leukoencephalopathy with spheroids^47–49^. Here we describe beneficial effects of peripheral Csf1 application to induce CSF1R signaling in non-PAM in an animal model for AD. Earlier studies suggested disease improvement in other brain disorders by application of IL-34 or Csf1 (refs. ^50–53^). A recent study highlighted protective effects of IL-34 application during autoimmune neuroinflammation in aged mice by expanding autophagy-dependent neuroprotective microglia^50^. Our data support the hypothesis that CSF1R engagement by Csf1 critically modulates microglial subsets and transcriptional states in amyloid pathology by reshaping the functionality of the non-PAM–PAM axis, reducing clonal expansion around the amyloid plaque and enhancing phagocytic activity. These results are in line with a recent report that showed that expansion of microglia in neurodegeneration after LPS treatment is driven by CSF1R signaling^37^. Our data point to a beneficial effect of Csf1 on controlling non-PAM-to-PAM differentiation and modulating PAM effector functions toward an amyloid-competent phenotype. Specifically, linear regression analyses via Voronoi gridding highlighted that microglial clonal expansion and amyloid deposition were highly correlated after Csf1 treatment. This approach can be used to dissect this correlation in future investigations on amyloid pathologies, although clonal relationships between microglia must be adequately established (for example, via fate mapping). Single-cell transcriptomic profiling further revealed an anti-inflammatory effect by Csf1 treatment and an enhancement of oxidative phosphorylation and reduced glycolysis, pointing to a metabolic switch in microglia, which was reported to be beneficial during amyloid pathology^54^.

In summary, our data point to the targeting of the dynamic transition of non-PAM to PAM as a thus-far-neglected key modulator of amyloid pathology and disease-associated microglial dynamics. We revealed that non-PAM are amyloid responsive, with individual non-PAM immediately responding to amyloid pathology by clonal expansion and differentiation to PAM. This transition can be modulated during early stages of amyloid pathology, providing a potential window of microglia subset-specific therapeutic intervention by adapting the transition from non-PAM to PAM.

## Methods

### Mice

Female *5×FAD* (*Tg6799*), *C57Bl/6J*, *Cx3cr1*^*CreERT2/+*^*R26*^*Confetti/+*^, *Tmem119*^*CreERT2/+*^*R26*^*Confetti*^, *Cx3cr1*^*CreERT2/+*^*R26*^*Confetti/+*^*5×FAD*^*+*^ and *Tmem119*^*CreERT2/+*^*R26*^*Confetti/+*^*5×FAD*^*+*^ mice were used in this study^55–57^. All animals were maintained in a specific pathogen-free facility with food and water ad libitum. For *Cx3cr1*^*CreERT2/+*^*R26*^*Confetti/+*^*5×FAD*^*+*^ mice, 5 mg of TAM (10 mg ml^−1^ in corn oil) was applied subcutaneously. For scRNA-seq and ATAC–seq experiments on *Cx3cr1*^*CreERT2/+*^*R26*^*Confetti/+*^*5×FAD*^*+*^ mice and all experiments on *Tmem119*^*CreERT2/+*^*R26*^*Confetti*^*5×FAD*^*+*^ mice, 10 mg of TAM (40 mg ml^−1^ in corn oil) was applied subcutaneously. Animal protocols were approved by the regional councils of Freiburg, Germany, and performed in accordance with the respective national, federal and institutional regulations.

### LPS treatment

Mice were intraperitoneally injected with 1 mg kg^−1^ LPS (Sigma-Aldrich, L3129) dissolved in a final concentration of 0.1 mg ml^−1^ in PBS twice a week for 8 weeks.

### ABX treatment

Specific pathogen-free mice were provided with drinking water containing 1 mg ml^−1^ cefoxitin (Santa Cruz Biotechnology), 1 mg ml^−1^ gentamicin (Sigma-Aldrich), 1 mg ml^−1^ metronidazole (Sigma-Aldrich) and 1 mg ml^−1^ vancomycin (Hexal) for 8 weeks ad libitum. Antibiotics were renewed every other day in light-protected water bottles.

### Csf1 and IL-34 treatment

Mice were intraperitoneally injected twice per week with 40 μg kg^−1^ recombinant murine Csf1 (PeproTech, 315-02) in PBS and 100 μg kg^−1^ IL-34 (BioLegend, 577602) in PBS or PBS alone for 8 weeks. For scRNA-seq analysis of Csf1-treated mice, all animals were treated for 4 weeks.

### Administration of BrdU

BrdU at a concentration of 0.1 mg g^−1^ (Sigma-Aldrich, B5002) (8 mg ml^−1^ in sterile PBS) was injected intraperitoneally twice a day over 5 days.

### Immunohistochemistry

Mice were anesthetized (intraperitoneal 100 mg of ketamine and 5 mg of xylazine per kilogram of body weight) and transcardially perfused with PBS. Brains were dissected and postfixed for 4–6 hours at 4 °C in 4% paraformaldehyde, washed with PBS and dehydrated in 30% sucrose at 4 °C. Samples were embedded and frozen in O.C.T. (Tissue-Tek). For all experiments, 60-µm sagittal brain sections were obtained with a cryostat (Leica), with the following exceptions, which were conducted with 30-µm sagittal brain sections: BrdU (Fig. 1c; see below); APOE, CLEC7A, AXL and P2RY12 (Fig. 2a and Extended Data Fig. 1); CD11c (Fig. 2b and Extended Data Fig. 1); and CD68 (Fig. 6e and Extended Data Fig. 2c). Sections were washed in wash buffer (WB; 0.1% Triton X-100 in PBS) before permeabilization in blocking buffer (BB; 0.5% Triton X-100, 5% BSA, 5% normal donkey serum, 0.1% NaN_3_ in PBS) for 1 hour on a shaker at room temperature. Primary antibodies were dissolved in BB and incubated overnight at 4 °C, including 1:200 anti-Pu.1 (Cell Signaling Technology, 2258S); 1:1,000 anti-GFP (Abcam, ab13970); 1:100 anti-P2RY12 (AnaSpec, 55043A); 1:100 anti-Clec7a (InVivoGen, mabg-mdect); 1:50 anti-CD11c (Novus Biologicals, NB110-97871); 1:500 anti-Iba-1 (Abcam, ab178846, or Wako, 019-19741); 1:200 anti-Iba-1 (Novus Biologicals, NB100-1028); 1:500 anti-Tmem119 (Abcam, ab209064); 1:100 anti-CD68 (Bio-Rad, MCA1957); 1:200 anti-Apoe (Merck, AB947); and 1:100 anti-Axl (R&D Systems, AF854). After incubation with primary antibodies, tissues were washed six times with WB. Secondary antibodies were diluted 1:1,000 in BB and incubated for 2 hours at room temperature, including donkey anti-rabbit Alexa Fluor 488 (Invitrogen, A21206), donkey anti-rabbit Alexa Fluor 568 (Invitrogen, A10042), donkey anti-rabbit Alexa Fluor 647 (Invitrogen A-31573), donkey anti-goat Alexa Fluor 647 (Life Technologies, A21447), chicken anti-rat Alexa Fluor 647 (Invitrogen, A21472), donkey anti-hamster Alexa Fluor 647 (Invitrogen, A21451) and donkey anti-chicken Alexa Fluor 488 (Jackson ImmunoResearch Europe Ltd., 703-545-155). Tissues were again washed six times with WB. Aβ plaques were stained with Methoxy-X04 (Tocris Bioscience, 4920) (30 µg ml^−1^), thioflavine-S (Sigma-Aldrich, T1892) (0.01%) and thiazine red (Morphisto, 12990) (0.01%). Methoxy-X04 (dilution 1:4), thioflavine-S (dilution 1:1,400) or thiazine red (dilution 1:20) was incubated for 10 minutes after antibody staining. After washing, sections were mounted with ProLong Diamond Antifade Mountant (Life Technologies, P36961).

For BrdU staining (Fig. 1c), 30-μm mouse brain sagittal sections were permeabilized as described before, followed by DNA denaturation in 2 M HCl for 18 minutes at 37 °C. After re-equilibration in PBS (pH 8.5), samples were blocked for 60 minutes at room temperature with BB before overnight incubation with 1:15 anti-BrdU antibody (Roche, 11170376001) at 4 °C. After washing with PBS, samples were incubated overnight with 1:500 anti-GFP (Abcam, ab13970) and 1:1,500 anti-RFP (Rockland, 600-401-379) at 4 °C. Alexa Fluor 568–conjugated donkey anti-mouse IgG (Life Technologies, A-10037), Alexa Fluor 488–conjugated donkey anti-chicken (Jackson ImmunoResearch Europe Ltd., 703-545-155) and Alexa Fluor 647–conjugated donkey anti-rabbit IgG (Invitrogen, A-31573) were used at 1:1,000, together with nuclear counterstain DAPI (1:5,000, Sigma-Aldrich) for 2 hours on a gentle shaker at room temperatiure. Sections were then mounted as described above.

### Microscopy

All immunofluroescent images were taken on a Leica SP8 confocal microscope or a Leica SP8X with white light laser, using a ×20, glycerine immersion, 0.95 numerical aperture (NA) W lens at a resolution of 1,024 × 1,024 pixels and *z*-step size between 1 µm and 1.04 µm. The following images were acquired on a Keyence BZ-9000 using a ×20, 0.75 NA objective lens: BrdU (Fig. 1c); APOE, CLEC7A and P2RY12 (Fig. 2a); CD11c (Fig. 2b); CD68 (Fig. 6e); APOE, CD11C, CLEC7A, AXL and P2RY12 (Extended Data Fig. 1); and CD68 (Extended Data Fig. 2c).

### Image analysis and quantification

Microglia labelling (for example, Confetti^+^, Pu.1^+^ and/or Iba-1^+^) and other quantifications (for example, number of Methoxy-X04^+^ plaques) were conducted through the open-source KNIME Analytics Platform (KNIME AG). Expression of proteins (for example, Tmem119; Fig. 2d) in microglia was established by overlaying fluorescent signal of interest to previously identified microglial cell bodies (for example, Iba-1^+^). Volumes of Methoxy-X04^+^ plaques were calculated by multiplying the sum of signal-positive pixels by the known image voxel dimensions. Analyzed images were then converted to HDF5 via the ImageJ Bioformats analyzer and HDF5 plugins, in order to quality control KNIME-generated quantifications with the iRoCS (Interactive Arabidopsis Root Analysis; Computer Vision Group Freiburg, https://lmb.informatik.uni-freiburg.de/resources/opensource/iRoCS/) Toolbox. PAM were defined with their processes in direct physical contact to Methoxy-X04-labeled amyloid deposits and their cell bodies within a 30-µm radius of said deposits or non-PAM as ramified cells distributed at greater distance from—and with no contact to—deposited extracellular amyloid. The area or volume, as appropriate, was then calculated for each analyzed image and used to normalize quantifications where relevant.

### Voronoi gridding—visualization or linear regression analysis (plaque size and microglial clone size)

Center coordinates (that is, centroids/Voronoi seeds) of microglia cell bodies (for example, Confetti^+^, Pu.1^+^ and/or Iba-1^+^) and Methoxy-X04^+^ plaques calculated via KNIME and iRoCS were *z*-projected into a single plane to compute a two-dimensional Voronoi mesh in RStudio (version 4.4.0) using the ‘bleiglas’ package (https://github.com/nevrome/bleiglas), Voro++ (https://math.lbl.gov/voro++/) and their associated dependencies. Voronoi cells of microglia were colored based on their fluorescence signal or combination of fluorescence signals, and Voronoi cells of Methoxy-X04^+^ plaques were colored based on their volume. As a spatial analysis tool to establish contact relationships between Voronoi cells, the resulting tessellation was used to quantify the size of Confetti^+^ microglia clones as well as to determine clone-to-plaque contacts. Clone-to-plaque contacts were used to establish linear regressions between microglia clone size and Aβ plaque size (Figs. 1h and 6n) and number of clones in contact to a given plaque (Fig. 6m). Voronoi grids were also used as a visual aide to represent the distribution of a given marker among microglia (that is, Tmem119 among Iba-1^+^or Iba-1^+^Confetti^+^ cells; Fig. 2d). In this use-case, PAM and non-PAM status was defined for microglia centroids based on plaque contact as observed in original fluorescent images, prior to grid generation and related quantification (Fig. 2e).

### MC simulation

The number of Confetti^+^ cells that shared the same color label was quantified through repeated measures taken from 10 µm to 290 µm (Figs. 1, 3 and 6) or from 10 µm to 190 µm (Extended Data Fig. 5). Starting from Confetti^+^ microglia, a 10-µm-radius ring was defined, and the number of same-colored microglia within that volume (10 µm *xy* × height of image in *z*) was recorded. After this, the ring radius was increased by 20 µm, quantifying the number of same-colored microglia within the 10–30-µm ring. This process was repeated until 270–290-µm radius from the origin cell. Calculations were performed on multiple images per biological replicate, before being averaged per replicate and then averaged by experimental group. After this, an MC simulation was performed, to test for clonal expansion or random recombination. During this simulation, the location of all Confetti^+^ cells is shuffled by switching the location of a Confetti^+^ cell with the location of a random Pu.1^+^ cell. After 10,000 shuffling simulations and quantifications, a baseline was established, which was then used to represent random distribution of Confetti labeling—that is, the randomized dataset. The 98% confidence interval was calculated for both the experimental and the randomized dataset. At distances where these confidence intervals do not overlap, the null hypothesis—that the Confetti labeling seen in the image is a result of random recombination—is rejected with a significance of *P* < 0.02. A full mathematical description is provided elsewhere^22^.

### ELISA

For the quantification of soluble and insoluble, Aβ40 and Aβ42 species, tissue from the frontal cortex was homogenized (10% w/v) in PBS + protease inhibitor and sequentially extracted with PBS (soluble fraction), with PBS + 0.1% Triton X-100 (membrane-bound fraction) and, finally, with 8 M guanidine hydrochloride solution. Protein concentration was measured with Bradford reagent (Roth), and ELISA was performed using an Amyloid Beta 42 Human ELISA Kit (Thermo Fisher Scientific, KHB3441) and an Amyloid Beta 40 Human ELISA Kit (Thermo Fisher Scientific, KHB3481) according to the manufacturer’s protocol.

### Western blot

Total protein from frontal cortex was extracted in RIPA buffer (50 mM HEPES, pH 7.5, 150 mM NaCl, 1 mM EDTA, 10% glycerin, 1% Triton X-100, 10 mM Na_4_O_7_P_2_, protease inhibitor). Protein concentration was measured with Bradford reagent (Roth). Samples were separated by 4–12% NuPAGE Bis-Tris mini gels using NuPAGE LDS sample buffer, NuPAGE sample reducing agent and NuPAGE MES SDS running buffer (Invitrogen). Proteins were transferred on PVDF membranes (Bio-Rad) and visualized using Clarity Western ECL Substrate (Bio-Rad). Antibodies against APP and C-terminal fragments (CTFs) (rabbit polyclonal antibody against the APP C terminus, 6687, 1:1,000), anti-Aβ (mouse, 1:3,000; Covance, 6E10) and anti-β-actin-HRP (mouse, 1:5,000; Abcam, ab20272) were used.

### Cell sorting

Index sorting was done using a MoFlo Astrios EQ at the Lighthouse Core Facility. The following antibodies and dyes were used: 1:500 Fixable Viability Dye in eFluor780 (Thermo Fisher Scientific, 65-0865-14); 1:200 for all antibodies used for the Dump channel in APC-Cy7 (CD3 clone 145-2C11, BioLegend, 100330; Gr1 clone RB6-8C5, BioLegend, 108423; CD19 clone 1D3, BD Biosciences, 557655); 1:100 for CD45 in BV786 (clone 30-F11, BD Biosciences, 564225); 1:100 for CD11b in BV605 (clone M1/70, BioLegend, 101257); 1:100 for CD11c in PE-Cy7 (clone N418, eBioscience, 25-0114-82); and 1:100 for Clec7a in APC (clone 17-5859-80, eBioscience, bg1fpj).

### scRNA-seq

Single cortical microglia were sorted into 384-well plates. The gating strategy is provided in Extended Data Fig. 3a. Cells were spun down and stored at −80 °C until further processing. RNA from single cells was isolated and transcribed into cDNA. For RNA sequencing, the mCEL-Seq2 protocol was used^58,59^.

### scRNA-seq analysis

Analysis of all three datasets was performed according to the following initial pipeline. The count matrices were analyzed with RStudio (version 2022.07.1 Build 554) using the Seurat package (version 5). Duplets were excluded by selecting cells with fewer than 4,000 nFeatures, and ambient noise was filtered by selecting cells with more than 200 nFeatures. The data were normalized using the ‘NormalizeData’ function with the ‘LogNormalize’ method; variable features were found using the ‘FindVariableFeatures’ function with the ‘variance stabilizing transformation’ method and selecting the top 4,000 features. Data were scaled using the ‘ScaleData’ function and then reduced in dimensions using PCA with the ‘RunPCA’ function.

For the scRNA-seq analysis of the early-stage dataset (Fig. 3 and Extended Data Fig. 3g–f), 1,095 cells remained after initial quality control and were embedded in a Euclidean space using the ‘RunUMAP’ function and the first 15 principal components. For the analysis of the late-stage dataset (Extended Data Fig. 3h–n), 1,017 cells were embedded as described above using the first 20 principal components and a clustering resolution of 1.0. For the CSF1-treated scRNA-seq dataset, 2,686 cells were embedded using the first five principal components at a clustering resolution of 0.7. Unsupervised clustering of the cells was done running the ‘FindNeighbors’ function for the first 15 dimensions and the ‘FindClusters’ function with a cluster resolution of 1.0 using the Louvain algorithm. The cluster resolution was selected after analyzing all possible ramifications using the ‘Clustree’ function from 0.1 to 1.5, with steps every 0.1. Differential abundance tests were calculated using a one-sided proportion test.

The heatmap of differentially expressed genes in the individual cell clusters was produced using the ‘DoHeatmap’ function, and the features were selected after calculating the top differentially expressed genes using a Wilcoxon test with the ‘FindAllMarkers’ function and an average log_2_ fold change (FC) threshold of 0.1. Genes with the following regular expressions were filtered out using ‘grepl’: ‘Gm\\d\\d’, ‘G\\d\\d’, ‘\\d\\d\\d’ and ‘mt-’. The dataset was further grouped by cluster, and the top 20 differentially expressed genes were selected for each cluster using the ‘top_n’ function and ordered by avg_log_2_ FC. The microglial activation signature was produced using ‘AddModuleScore’ and ‘FeaturePlot’^11^. The homeostatic microglia gene signature was calculated^11^. The expression of gene expression modules was quantified using the UCell R package version 2.4.0. The gene lists associated with Gene Ontology terms presented in Fig. 4 were obtained from the object MSigDB_v6.0_C5_mouse within the MOFAdata R package version 1.16.1. The module enrichment scores quantified by the Mann–Whitney *U* statistic were visualized using the ‘VlnPlot’ Seurat function.

GSEA was done using GSEA 4.3.2 software^60^. The ranked list of the gene name and avg_log_2_ FC for the GSEA was obtained by using the ‘FindMarkers’ function with the Model-based Analysis of Single-cell Transcriptomics (MAST) test^61^, setting a log FC threshold of 0 and a min.pct of 0.001.

Volcano plots were produced using the ‘EnhancedVolcano’ function, with the avg_log_2_ FC on the *x* axis and the p_val_adj on the y axis, an FC cutoff of 0.25 and an adjusted *P* value cutoff of 0.05.

### ATAC–seq

To profile chromatin accessibility, the ATAC–seq protocol was performed. In total, 50,000 microglia were sorted in 250 μl of FACS buffer (1× PBS, 2% FBS, 2 mM EDTA) and pelleted by centrifugation for 5 minutes, 500*g* at 4 °C. Cell pellets were washed with 1× PBS and pelleted by centrifugation. Cell pellets were resuspended in 50 μl of cold lysis buffer (10 mM Tris-HCl, pH 7.4, 10 mM NaCl, 3 mM MgCl_2_, 0.1% IGEPAL CA-630). Nuclei were pelleted by centrifugation for 10 minutes, 500*g* at 4 °C, and resuspended in 50 μl of reaction buffer containing 2.5 μl of Tn5 transposase and 25 μl of TD buffer (Nextera sample preparation kit; Illumina). The reaction was incubated at 37 °C for 45 minutes with gentle mixing. Afterwards, tagmented DNA was purified using a MinElute PCR Purification Kit (Qiagen) according to the manufacturerʼs instructions. Libraries were initially amplified by a five-cycle PCR before each sample was assessed by RT–qPCR for the optimum number of extra PCR amplification cycles (maximum, nine) to reduce GC and size bias. Then, 5 μl of indexing primers and NEBNext Q5 Hot Start HiFi PCR Master Mix (New England Biolabs) was used. Library cleanup was performed twice using NucleoMag NGS Beads (Macherey-Nagel). DNA concentration was measured with a Qubit fluorometer (Life Technologies). Library sizes and quality were determined using Bioanalyzer (Agilent Technologies). Libraries were sequenced on an Illumina HiSeq 2000 for an average of 30 million unique reads per sample.

### ATAC–seq analysis

Reads were aligned to the mouse reference genome (GRCm38/mm10) using Bowtie 2 (version 2.3.4.3)^62^. Adaptors and low-quality read ends were trimmed using Trim Galore (version 0.4.3.1, https://github.com/FelixKrueger/TrimGalore). Mitochondrial DNA reads were removed with BAMTools (version 2.5.1)^63^, and blacklisted genomic regions for mm10 defined by ENCODE^64^ were excluded using ‘bedtools intersect’ (version 2.3.0.0)^65^. For quality control, transcription start site enrichment (TSSe) scores were calculated, and samples with TSSe less than 15 were removed from downstream analysis according to ENCODE standards (https://www.encodeproject.org/data-standards/atac-seq/atac-encode4/). Peaks were called with MACS2 (version 2.1.1.20160309.6, –shift -100 –extsize 200 –nomodel –call-summits -q 0.05)^66^. Peaks from individual samples were combined into a consensus peak list. DARs were identified using DESeq2 (version 2.11.40.7, log_2_ FC ≥ 0.584 or log_2_ FC ≤ −0.584, false discovery rate (FDR) < 0.05)^67^, followed by region annotation using ChIPSeeker (version 1.18.0). DeepTools2 (version 3.5.1) was used for visualization^68^. Individual sample bigwig files were normalized with DESeq2 size factors before being merged with bigwigAverage^68^. DeepTools2 (version 3.5.1, computeMatrix, plotHeatmap) was used to visualize chromatin accessibility from merged bigwig files at defined microglia-specific primed and active enhancer regions^69^. Motif enrichment analysis was performed using HOMER (version 4.11)^70^. Pathway enrichment analysis was performed using GREAT (version 4.04)^71^. The analysis workflow and visualizations were performed on the Galaxy platform^72^.

### Statistics and reproducibility

Quantifications were performed in a blinded manner by assignment of unidentifiable numbers to mice, tissues and images. All experiments were performed at least twice, except as otherwise indicated in the figure legends. scRNA-seq and ATAC–seq experiments were performed only as individual experiments. No statistical methods were used to predetermine sample sizes. GraphPad Prism 9 was used for statistical testing. Datasets were tested for homoscedasticity (Brown–Forsythe test) and normality (D’Agostino–Pearson omnibus, Anderson–Darling, Shapiro–Wilk and Kolmogorov–Smirnov) to determine the appropriate statistical test. Statistical outlier test was performed before including samples in the statistical analysis. The following statistical tests were used: Mann–Whitney test (Figs. 2k and 6g,l,m and Extended Data Fig. 5d,g); Kruskal–Wallis test with Dunn’s post hoc testing (Fig. 3c,g–i and Extended Data Fig. 2a); ordinary one-way ANOVA with Tukey post hoc testing (Fig. 3e and Extended Data Fig. 2b,d); unpaired *t*-test (Figs. 2e and 6b–d,f,g,k and Extended Data Fig. 5d–g); Kolmogrov–Smirnov test (Fig. 6l); Brown–Forsythe and Welch ANOVA with Dunnett’s T3 multiple comparisons test (Extended Data Fig. 2a); and linear regression (Figs. 1h and 6n). Differences were considered statistically significant at *P* *<* 0.05. Data are presented as mean ± s.e.m. unless indicated otherwise.

### Reporting summary

Further information on research design is available in the Nature Portfolio Reporting Summary linked to this article.

## Online content

Any methods, additional references, Nature Portfolio reporting summaries, source data, extended data, supplementary information, acknowledgements, peer review information; details of author contributions and competing interests; and statements of data and code availability are available at 10.1038/s41593-025-02006-0.

## Supplementary information

Reporting Summary

Supplementary Video 1 Representative video of a three-dimensional reconstruction from frontal cortices in adult *Tmem119*^*CreERT2*^*R26*^*Confetti*^*5×FAD*^*+*^ animals 2 weeks after TAM application, as presented in Fig. 2j. Iba-1 was removed from the reconstruction to increase visibility of the Confetti-labeled clone. Immunofluorescence for Methoxy-X04 (Aβ, blue) and mCFP (cyan) is shown.

Supplementary Video 2 Representative video of a three-dimensional reconstruction from frontal cortices in adult *Tmem119*^*CreERT2*^*R26*^*Confetti*^*5×FAD*^*+*^ animals 8 weeks after TAM application, as presented in Fig. 2j. Iba-1 was removed from the reconstruction to increase visibility of the Confetti-labeled clone. Immunofluorescence for Methoxy-X04 (Aβ, blue) and YFP (yellow) is shown.

Supplementary Table 1 Chromatin accessibility differences between PAM and non-PAM.

## Source data

Source Data Fig. 6h Uncropped western blot images of Fig. 6h. The following images are shown: anti-APP (**a**), anti-Aβ_1–16_ (**b**), anti-β-actin (**c**) and colorimetric protein ladder (**d**).

Source Data Figs. and Extended Data Fig. Individual data points are shown of all quantifications shown in this study. This includes the quantifications shown in Figs. 1b,c,h, 2e,k,l, 3g–i and 6c,d,f,g,k,l–n and Extended Data Figs. 2a,b,d and 5e–g.

Source Data Figs. and Extended Data Fig. Data tested in MC simulations shown in Figs. 1f, 3c,e and 6b and Extended Data Fig. 5d.

## Data Availability

The raw and processed data for this project are uploaded to the Gene Expression Omnibus database and are accessible under the following accession numbers: GSE296025 (ATAC–seq), GSE296026 (scRNAseq1_LPS_ABX) and GSE296027 (scRNAseq2_Csf1). Source data are provided with this paper.

## Code availability

The computer code for the scRNA-seq analysis within this project can be found and explored at https://www.brain-immunity.de/. No custom code was developed for the workflow for the ATAC–seq analysis. Established methods were used for bioinformatic analysis and are cited in the main text and Methods.
