# Supplementary material for: Response of spatially defined microglia states with distinct chromatin accessibility in a mouse model of Alzheimer’s disease
Source: Nat Neurosci. 2025 Jul 14;28(8):1688–703. doi: 10.1038/s41593-025-02006-0 (PMC12321583; doi:10.1038/s41593-025-02006-0)
Supplement: Supplementary file 1 — Reporting Summary [file 41593_2025_2006_MOESM1_ESM.pdf]

nature portfolio

Corresponding author(s): Marco Prinz

Last updated by author(s): May 1, 2025

Reporting Summary

Nature Portfolio wishes to improve the reproducibility of the work that we publish. This form provides structure for consistency and transparency in reporting. For further information on Nature Portfolio policies, see our Editorial Policies and the Editorial Policy Checklist.

Statistics

For all statistical analyses, confirm that the following items are present in the figure legend, table legend, main text, or Methods section.

n/a	Confirmed	
☐	☒	The exact sample size (n) for each experimental group/condition, given as a discrete number and unit of measurement
☐	☒	A statement on whether measurements were taken from distinct samples or whether the same sample was measured repeatedly
☐	☒	The statistical test(s) used AND whether they are one- or two-sided<br>*Only common tests should be described solely by name; describe more complex techniques in the Methods section.*
☐	☒	A description of all covariates tested
☐	☒	A description of any assumptions or corrections, such as tests of normality and adjustment for multiple comparisons
☐	☒	A full description of the statistical parameters including central tendency (e.g. means) or other basic estimates (e.g. regression coefficient) AND variation (e.g. standard deviation) or associated estimates of uncertainty (e.g. confidence intervals)
☐	☒	For null hypothesis testing, the test statistic (e.g. F, t, r) with confidence intervals, effect sizes, degrees of freedom and P value noted<br>*Give P values as exact values whenever suitable.*
☒	☐	For Bayesian analysis, information on the choice of priors and Markov chain Monte Carlo settings
☒	☐	For hierarchical and complex designs, identification of the appropriate level for tests and full reporting of outcomes
☐	☒	Estimates of effect sizes (e.g. Cohen's d, Pearson's r), indicating how they were calculated

Our web collection on statistics for biologists contains articles on many of the points above.

Software and code

Policy information about availability of computer code

Data collection	LAS X software was used for confocal imaging with the Leica TCS SP8 X<br><br>BZ-II Viewer software was used for the Keyence BZ-9000 inverted fluorescence microscope.<br><br>Summit v6.3.1 software was used for the sorting on the MoFlo Astrios EQ.<br><br>HiSeq Control Software 2.0.2, RTA 2.4.11 / Recipe Fragment 2.0.0.2 software was used for image aquisition, intensity extraction and basecalling on the HighSeq 3000 sequencer<br><br>NovaSeq Control Software was used on the Nova6000 sequencer<br>Demultiplexing was perfomed with Illumina's bcl2fastq2 software (https://support.illumina.com/downloads/bcl2fastq-conversion-software-v2-20.html), and the version 2.20.0.422).
Data analysis	GraphPad Prism v9 was used for graph design and statistical analysis.<br>KNIME software (KNIME AG, Zurich, Switzerland) for cell labeling and quantification through a custom-designed image analysis algorithm.<br><br>ImageJ Bioformats analyzer & HDF5 plug ins to save the files in HDF5 format.<br><br>iRoCS toolbox (version 1.2.3, open source software, Computer Vision Group, Freiburg) to supervise and correct labelings done by the algorithms designed in KNIME.<br><br>FlowJo software, version 10.7 was used to process FACS data.<br><br>mCEL-Seq2 protocol was used for RNA sequencing (10.1186/s13059-016-0938-8)

bwa (version 0.6.2-r126) with default parameters was used for quantification of transcript abundance using paired-end reads aligned to the transcriptome as described in (10.1126/science.aat7554).

The RaceID algorithm was used for scRNA sequencing data analysis (10.1038/nature14966).

StemID2 and FateID (10.1016/j.stem.2016.05.010 & 10.1038/nmeth.4662) were used for conducting Pseudotime analysis.

```
Data analysis and visualization for scRNA-seq was conducted via R version 4.2.2 (2022-10-31)
Platform: aarch64-apple-darwin20 (64-bit)
Running under: macOS Ventura 13.4.1

Matrix products: default
LAPACK: /Library/Frameworks/R.framework/Versions/4.2-arm64/Resources/lib/libRlapack.dylib

locale:
[1] en_US.UTF-8/en_US.UTF-8/en_US.UTF-8/C/en_US.UTF-8/en_US.UTF-8

attached base packages:
 [1] splines   grid      stats4    stats     graphics  grDevices utils     datasets  methods   base

other attached packages:
 [1] describedata_0.1.0          zoo_1.8-12                  SingleR_2.0.0
 [4] monocle_2.26.0              DDRTree_0.1.5               irlba_2.3.5.1
 [7] VGAM_1.1-8                  Matrix_1.5-4.1              EnhancedVolcano_1.16.0
[10] ggrepel_0.9.3               ggsignif_0.6.4              clustree_0.5.0
[13] ggraph_2.1.0                msigdbr_7.5.1               fgsea_1.24.0
[16] DESeq2_1.38.3               data.table_1.14.8           gtable_0.3.3
[19] gridExtra_2.3               SeuratObject_4.1.3          Seurat_4.3.0
[22] lubridate_1.9.2             forcats_1.0.0               stringr_1.5.0
[25] dplyr_1.1.2                 purrr_1.0.1                 readr_2.1.4
[28] tidyr_1.3.0                 tibble_3.2.1                ggplot2_3.4.2
[31] tidyverse_2.0.0             harmony_0.1.1               Rcpp_1.0.10
[34] RColorBrewer_1.1-3          scales_1.2.1                monocle3_1.3.1
[37] SingleCellExperiment_1.20.1 SummarizedExperiment_1.28.0 GenomicRanges_1.50.2
[40] GenomeInfoDb_1.34.9         IRanges_2.32.0              S4Vectors_0.36.2
[43] MatrixGenerics_1.10.0       matrixStats_1.0.0           Biobase_2.58.0
[46] BiocGenerics_0.44.0

loaded via a namespace (and not attached):
  [1] rappdirs_0.3.3            scattermore_1.1           bit64_4.0.5
  [4] knitr_1.43                DelayedArray_0.24.0       KEGGREST_1.38.0
  [7] RCurl_1.98-1.12           generics_0.1.3            ScaledMatrix_1.6.0
 [10] leidenbase_0.1.18         callr_3.7.3               terra_1.7-29
 [13] cowplot_1.1.1             usethis_2.1.6             RSQLite_2.3.1
 [16] RANN_2.6.1                combinat_0.0-8            future_1.32.0
 [19] bit_4.0.5                 tzdb_0.4.0                spatstat.data_3.0-1
 [22] httpuv_1.6.11             viridis_0.6.3             xfun_0.39
 [25] celldex_1.8.0             hms_1.1.3                 babelgene_22.9
 [28] evaluate_0.21             promises_1.2.0.1          fansi_1.0.4
 [31] dbplyr_2.3.2              igraph_1.4.3              DBI_1.1.3
 [34] geneplotter_1.76.0        htmlwidgets_1.6.2         sparsesvd_0.2-2
 [37] spatstat.geom_3.2-1       ellipsis_0.3.2            backports_1.4.1
 [40] annotate_1.76.0           deldir_1.0-9              sparseMatrixStats_1.10.0
 [43] vctrs_0.6.2               remotes_2.4.2             ROCR_1.0-11
 [46] abind_1.4-5               cachem_1.0.8              withr_2.5.0
 [49] ggforce_0.4.1             progressr_0.13.0          sctransform_0.3.5
 [52] prettyunits_1.1.1         goftest_1.2-3             cluster_2.1.4
 [55] ExperimentHub_2.6.0       lazyeval_0.2.2            crayon_1.5.2
 [58] spatstat.explore_3.2-1    pkgconfig_2.0.3           slam_0.1-50
 [61] tweenr_2.0.2              nlme_3.1-162              pkgload_1.3.2
 [64] devtools_2.4.5            rlang_1.1.1               globals_0.16.2
 [67] lifecycle_1.0.3           miniUI_0.1.1.1            filelock_1.0.2
 [70] BiocFileCache_2.6.1       rsvd_1.0.5                AnnotationHub_3.6.0
 [73] polyclip_1.10-4           lmtest_0.9-40             boot_1.3-28.1
 [76] ggridges_0.5.4            processx_3.8.1            pheatmap_1.0.12
 [79] png_0.1-8                 viridisLite_0.4.2         bitops_1.0-7
 [82] KernSmooth_2.23-21        Biostrings_2.66.0         blob_1.2.4
 [85] DelayedMatrixStats_1.20.0 parallelly_1.36.0         spatstat.random_3.1-5
 [88] beachmat_2.14.2           memoise_2.0.1             magrittr_2.0.3
 [91] plyr_1.8.8                ica_1.0-3                 zlibbioc_1.44.0
 [94] compiler_4.2.2            HSMMSingleCell_1.18.0     lme4_1.1-33
 [97] fitdistrplus_1.1-11       cli_3.6.1                 XVector_0.38.0
[100] urlchecker_1.0.1          listenv_0.9.0             patchwork_1.1.2
[103] pbapply_1.7-0             ps_1.7.5                  MASS_7.3-60
[106] tidyselect_1.2.0          stringi_1.7.12            yaml_2.3.7
```

```
[109] BiocSingular_1.14.0     locfit_1.5-9.7          fastmatch_1.1-3
[112] tools_4.2.2             timechange_0.2.0        future.apply_1.11.0
[115] parallel_4.2.2          rstudioapi_0.14         farver_2.1.1
[118] Rtsne_0.16              digest_0.6.31           BiocManager_1.30.20
[121] shiny_1.7.4             qlcMatrix_0.9.7         broom_1.0.4
[124] BiocVersion_3.16.0      later_1.3.1             RcppAnnoy_0.0.20
[127] httr_1.4.6              AnnotationDbi_1.60.2    colorspace_2.1-0
[130] XML_3.99-0.14           fs_1.6.2                tensor_1.5
[133] reticulate_1.28         uwot_0.1.14             spatstat.utils_3.0-3
[136] graphlayouts_1.0.0      sp_1.6-1                plotly_4.10.2
[139] sessioninfo_1.2.2       xtable_1.8-4            jsonlite_1.8.5
[142] nloptr_2.0.3            tidygraph_1.2.3         R6_2.5.1
[145] profvis_0.3.8           pillar_1.9.0            htmltools_0.5.5
[148] mime_0.12               glue_1.6.2              fastmap_1.1.1
[151] minqa_1.2.5             BiocParallel_1.32.6     interactiveDisplayBase_1.36.0
[154] codetools_0.2-19        pkgbuild_1.4.0          utf8_1.2.3
[157] lattice_0.21-8          spatstat.sparse_3.0-1   curl_5.0.0
[160] leiden_0.4.3            limma_3.54.2            survival_3.5-5
[163] docopt_0.7.1            rmarkdown_2.22          fastICA_1.2-3
[166] munsell_0.5.0           GenomeInfoDbData_1.2.9  haven_2.5.2
[169] reshape2_1.4.4
```

FastQC (v0.67) was used to check the quality of the raw data for the ATAC-seq.

Bowtie2 aligner (v2.3.4.3) was used to align the reads to the mouse reference genome for the ATAC-seq.

Trim Galore! (v0.4.3.1) was used to trim the adaptors and low quality read ends for the ATAC-seq.

The Picard tool MarkDuplicates (v2.18.2.2) was used to remove PCR duplicates for the ATAC-seq.

BAMTools (v2.5.1) was used to remove mitochondrial DNA for the ATAC-seq.

bedtools intersect (v2.3.0.0) was used to exclude blacklisted genomic regions for mm10 defined by ENCODE for the ATAC-seq.

DESeq2 (v2.11.40.7) was used to identify differentially accessible regions in ATAC-seq data analysis.

ChIPSeeker (v1.18.0) was used to perform region annotation for the ATAC-seq data.

DeepTools2 (Version 3.5.1) was used for visualization for the ATAC-seq data.

HOMER (v4.11) was used for motif enrichment analysis of the ATAC-seq data.

GREAT (v4.04) was used for pathway enrichment analysis of the ATAC-seq data.

The Galaxy platform (web-based, no version number available) was used to perform the whole ATAC-seq analysis workflow (see methods).

For manuscripts utilizing custom algorithms or software that are central to the research but not yet described in published literature, software must be made available to editors and reviewers. We strongly encourage code deposition in a community repository (e.g. GitHub). See the Nature Portfolio guidelines for submitting code & software for further information.

Data

Policy information about availability of data

All manuscripts must include a data availability statement. This statement should provide the following information, where applicable:

- Accession codes, unique identifiers, or web links for publicly available datasets
- A description of any restrictions on data availability
- For clinical datasets or third party data, please ensure that the statement adheres to our policy

All datasets generated within this study are available on the GEO database platform under the following accession numbers: GSE296025 (ATAC-seq), GSE296026 (scRNAseq1_LPS_ABX), GSE296027 (scRNAseq2_Csf1)

Field-specific reporting

Please select the one below that is the best fit for your research. If you are not sure, read the appropriate sections before making your selection.

☒ Life sciences ☐ Behavioural & social sciences ☐ Ecological, evolutionary & environmental sciences

For a reference copy of the document with all sections, see nature.com/documents/nr-reporting-summary-flat.pdf

Life sciences study design

All studies must disclose on these points even when the disclosure is negative.

Sample size	No statistical methods were used to predetermine sample sizes. Samples sizes were chosen to allow statistical testing and varied depending on the breeding capacity of the mouse lines used (litter sizes).
Data exclusions	All datasets were tested for statistical outliers.<br>For the ATAC-seq: Datasets with Transcriptional start site enrichment (TSSe) > 15 were used for downstream analysis of ATAC-seq datasets. Based on this threshold, the sample "Non-PAM rep2" was removed before downstream analysis (see Extended Data Figure 4)
Replication	To be sure of the reproducibility of the experimental findings, all experiments were replicated twice successfully, if not otherwise stated.
Randomization	For all experiments, mice were randomly allocated to each experimental group by AAF and LFPB.
Blinding	All quantification experiments were performed in a blinded manner by assignment of unidentifiable numbers to mice, tissues and images for data acquisition and processing. Data labels and groups were only reinstated for statistical analysis. Quantification and imaging was not repeated following statistical analysis.

Reporting for specific materials, systems and methods

We require information from authors about some types of materials, experimental systems and methods used in many studies. Here, indicate whether each material, system or method listed is relevant to your study. If you are not sure if a list item applies to your research, read the appropriate section before selecting a response.

Materials & experimental systems

n/a	Involved in the study
☐	☒ Antibodies
☒	☐ Eukaryotic cell lines
☒	☐ Palaeontology and archaeology
☐	☒ Animals and other organisms
☒	☐ Human research participants
☒	☐ Clinical data
☒	☐ Dual use research of concern

Methods

n/a	Involved in the study
☒	☐ ChIP-seq
☐	☒ Flow cytometry
☒	☐ MRI-based neuroimaging

Antibodies

Antibodies used	For histological analysis:<br>- Primary antibodies: Rabbit anti-Pu.1 (Cell Signaling, 2258S), chicken anti-GFP (Abcam ab13970), hamster anti-CD11c (Novus NB110-97871), rabbit anti-Iba-1 (Abcam ab178846 or Wako 019-19741), goat anti-Iba-1 (Novus NB100-1028), rabbit anti-Tmem119 (Abcam ab209064), rat anti-CD68 (Biorad MCA1957), rabbit P2RY12 (Anaspec 55043A), goat anti-ApoE (Merck AB947), goat anti-Axl (R&D systems AF854), mouse anti-BrdU antibody (Roche 11170376001), and rabbit anti-RFP (Rockland 600-401-379).<br>- Secondary antibodies: donkey anti-rabbit Alexa Fluor 488 (Invitrogen A21206), donkey anti-rabbit Alexa Fluor 568 (Invitrogen A10042), donkey anti-rabbit Alexa Fluor 647 (Invitrogen A-31573), donkey anti-goat Alexa Fluor 647 (Life Technologies A21447), chicken anti-rat Alexa Fluor 647 (Invitrogen A21472), donkey anti-hamster Alexa Fluor 647 (Invitrogen A21451) and donkey anti-Chicken Alexa Fluor 488 (Jackson ImmunoResearch Europe Ltd 703-545-155), donkey anti-mouse IgG (Life Technologies A-10037), Alexa Fluor 488-conjugated donkey anti-chicken (Jackson ImmunoResearch Europe Ltd 703-545-155) and Alexa Fluor 647-conjugated donkey anti-rabbit IgG (Invitrogen A-31573).<br><br>For cell sorting:<br>APCCy7 (CD3 clone 145-2C11 from BioLegend #100330, Gr1 clone RB6-8C5 from BioLegend #108423, CD19 clone 1D3 from BD #557655); for CD45 in BV786 (clone 30-F11 BD #564225); for CD11b in BV605 (clone M1/70 BioLegend #101257); for CD11c in PeCy7 (clone N418 eBioscience #25-0114-82); for Clec7a in APC (clone 17-5859-80 eBioscience #bg1fpj).<br><br>For ELISA:<br>The following kits (including primary antibodies) were used: E Amyloid beta 42 Human ELISA Kit (ThermoFischer Scientific, #KHB3441) and Amyloid beta 40 Human ELISA Kit (ThermoFischer Scientific, #KHB3481).<br><br>For western blot:<br>The follwing antibodies were used for western blot: anti-APP C-terminus (for APP and CTFs), (rabbit ,6687, 1:1000), anti-Aβ (mouse, 1:3000, Covance, 6E10) and anti-β-actin-HRP (mouse, 1:5000, abcam, ab20272).
Validation	All primary anti-mouse antibodies used in the flow cytometry, immunofluorescence , and immunoblotting have been validated for this application by the supplier as indicated on the websites and datasheets of the coording antibodies. Companies, order numbers

and/or clone names are indicated for all used antibodies.

Animals and other organisms

Policy information about studies involving animals; ARRIVE guidelines recommended for reporting animal research

Laboratory animals	Female 5xFAD, C57Bl/6J (wildtype), Cx3cr1creERT2/+R26RConfetti/+ and 5xFAD Cx3cr1creERT2/+R26RConfetti/+ and 5xFAD Tmem119CreERT2/+ R26RConfetti/+ mice were used in this study. All mice were bred in-house under specific pathogen-free conditions with food and water ad libitum (12:12 h light-dark cycle).
Wild animals	No wild animals were used in this study.
Field-collected samples	No wild animals were used in this study.
Ethics oversight	Animal studies were approved by the Regional Councils of Freiburg, Germany and performed in accordance to the respective national, federal and institutional regulations.

Note that full information on the approval of the study protocol must also be provided in the manuscript.

Flow Cytometry

Plots

Confirm that:

☒ The axis labels state the marker and fluorochrome used (e.g. CD4-FITC).

☒ The axis scales are clearly visible. Include numbers along axes only for bottom left plot of group (a 'group' is an analysis of identical markers).

☒ All plots are contour plots with outliers or pseudocolor plots.

☒ A numerical value for number of cells or percentage (with statistics) is provided.

Methodology

Sample preparation	Mice were anesthetized (i.p. 100 mg ketamine and 5 mg xylazine per kg body weight) and transcardially perfused with PBS. The brain was taken out and the cortex was manually dissected and placed in dissection media at 4C, then homogenized using a glass potter. The solution was passed through a 70µm cell strainer, then centrifuged at 200g for 5mins. The supernatant was removed and the pellet resuspended in 37% Percoll. After centrifuging at 800g for 30mins at 4°C, the myelin and pellet was removed. The pellet was resuspended, washed once in PBS, centrifuged, then resuspended in a staining solution for 1hr at 4C. The following antibodies and dyes were used: 1:500 Fixable Viability Dye in eFluor780 (Thermofisher 65-0865-14); 1:200 for all antibodies used for the dump channel in APCCy7 (CD3 clone 145-2C11 from BioLegend #100330, Gr1 clone RB6-8C5 from BioLegend #108423, CD19 clone 1D3 from BD #557655); 1:100 for CD45 in BV786 (clone 30-F11 BD #564225); 1:100 for CD11b in BV605 (clone M1/70 BioLegend #101257); 1:100 for CD11c in PeCy7 (clone N418 eBioscience #25-0114-82); 1:100 for Clec7a in APC (clone 17-5859-80 eBioscience #bg1fpj).
Instrument	Cells were sorted using a MoFlo Astrios EQ (Beckman Coulter) or analyzed using a BD LSRFortessa (Becton Dickinson).
Software	Data were acquired with FACSDiva software (Becton Dickinson). Postacquisition analysis was performed using FlowJo software, version 10.7
Cell population abundance	Cell population abundance was not tested in flow cytometry. FACS sorting was employed to purify PAM and non-PAM from different transgenic mice. The obtained cell number does not indicate cell abundance and no further analysis of cell abundance was performed via flow cytometry in this study.
Gating strategy	In all experiments, small debris was removed with the preliminary FSC/SSC gate. Single, living cells were obtained by doublet exclusion followed by the exclusion of dead cells using live-dead dyes.

☒ Tick this box to confirm that a figure exemplifying the gating strategy is provided in the Supplementary Information.
