# Supplementary material for: Response of spatially defined microglia states with distinct chromatin accessibility in a mouse model of Alzheimer’s disease
Source: Nat Neurosci. 2025 Jul 14;28(8):1688–703. doi: 10.1038/s41593-025-02006-0 (PMC12321583; doi:10.1038/s41593-025-02006-0)
Supplement: Supplementary file 5 — Uncropped western blot images of Fig. 6h. The following images are shown: anti-APP (a), anti-Aβ1–16 (b), anti-β-actin (c) and colorimetric protein ladder (d). [file 41593_2025_2006_MOESM5_ESM.pdf]

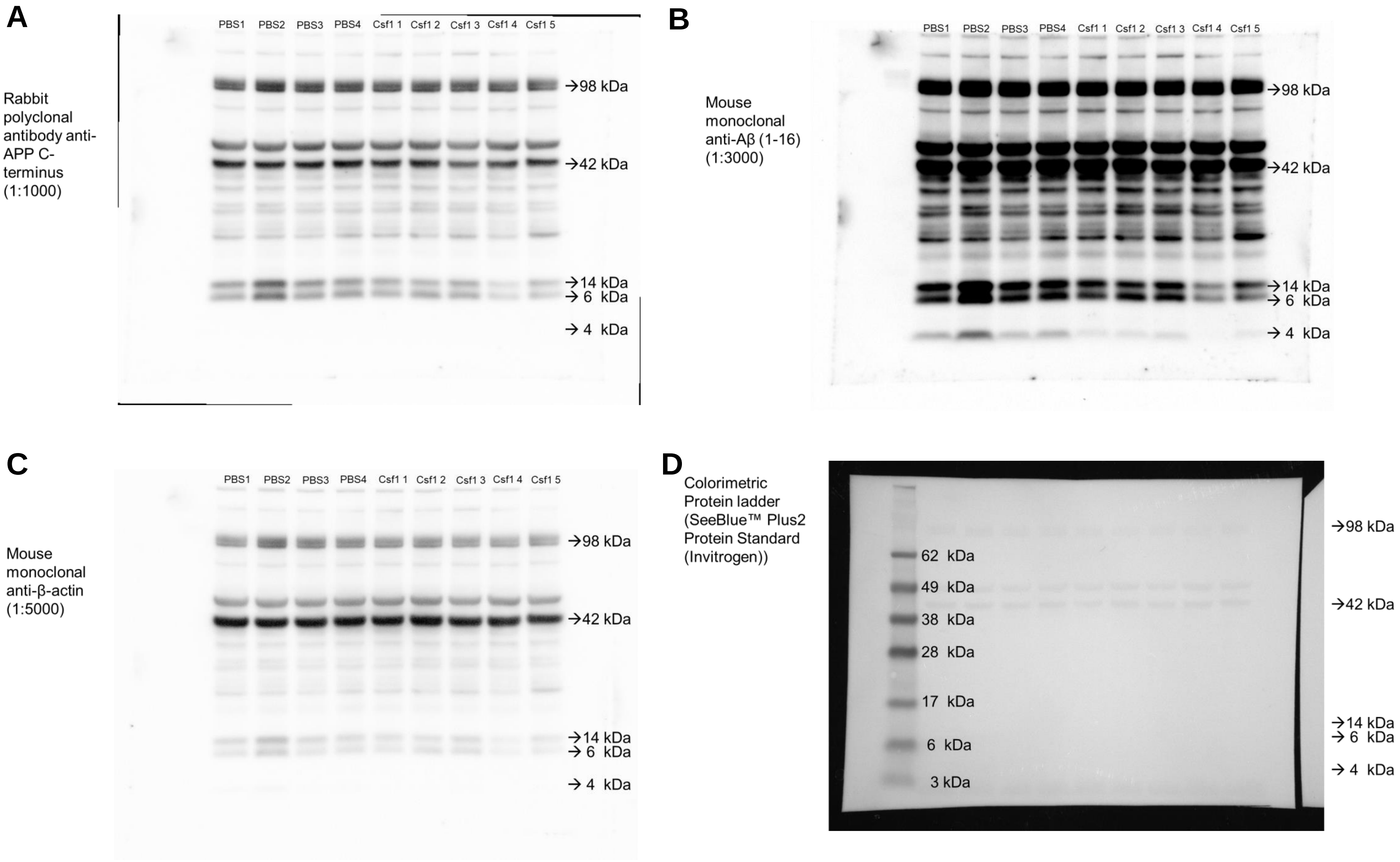
A
Rabbit polyclonal antibody anti-APP C-terminus (1:1000)
PBS1 PBS2 PBS3 PBS4 Csf1 1 Csf1 2 Csf1 3 Csf1 4 Csf1 5
→98 kDa
→42 kDa
→14 kDa
→ 6 kDa
→ 4 kDa
B
Mouse monoclonal anti-Aβ (1-16) (1:3000)
PBS1 PBS2 PBS3 PBS4 Csf1 1 Csf1 2 Csf1 3 Csf1 4 Csf1 5
→98 kDa
→42 kDa
→14 kDa
→ 6 kDa
→ 4 kDa
C
Mouse monoclonal anti-β-actin (1:5000)
PBS1 PBS2 PBS3 PBS4 Csf1 1 Csf1 2 Csf1 3 Csf1 4 Csf1 5
→98 kDa
→42 kDa
→14 kDa
→ 6 kDa
→ 4 kDa
D
Colorimetric Protein ladder (SeeBlue™ Plus2 Protein Standard (Invitrogen))
62 kDa
49 kDa
38 kDa
28 kDa
17 kDa
6 kDa
3 kDa
→98 kDa
→42 kDa
→14 kDa
→ 6 kDa
→ 4 kDa
